# First π-linker featuring mercapto and isocyano anchoring groups within the same molecule: synthesis, heterobimetallic complexation and self-assembly on Au(111)†
†Electronic supplementary information (ESI) available: Experimental procedures, spectroscopic and analytical data, details of the crystallographic and computational studies. CCDC 1410785. For ESI and crystallographic data in CIF or other electronic format see DOI: 10.1039/c5sc04017e


**DOI:** 10.1039/c5sc04017e

**Published:** 2015-11-20

**Authors:** Jason C. Applegate, Monisola K. Okeowo, Nathan R. Erickson, Brad M. Neal, Cindy L. Berrie, Nikolay N. Gerasimchuk, Mikhail V. Barybin

**Affiliations:** a Department of Chemistry , The University of Kansas , 1251 Wescoe Hall Drive , Lawrence , KS 66045 , USA . Email: mbarybin@ku.edu ; Email: cberrie@ku.edu; b Department of Chemistry , Missouri State University , 901 S. National Ave. , Springfield , MO 65897 , USA . Email: NNGerasimchuk@MissouriState.edu

## Abstract

Mercapto (–SH) and isocyano (–N≡C) terminated conducting π-linkers are often employed in the ever-growing quest for organoelectronic materials. While such systems typically involve symmetric dimercapto or diisocyano anchoring of the organic bridge, this article introduces the chemistry of a linear azulenic π-linker equipped with one mercapto and one isocyano terminus. The 2-isocyano-6-mercaptoazulene platform was efficiently accessed from 2-amino-6-bromo-1,3-diethoxycarbonylazulene in four steps. The 2-N≡C end of this 2,6-azulenic motif was anchrored to the [Cr(CO)_5_] fragment prior to formation of its 6-SH terminus. Metalation of the 6-SH end of [(OC)_5_Cr(η^1^-2-isocyano-1,3-diethoxycarbonyl-6-mercaptoazulene)] (**7**) with Ph_3_PAuCl, under basic conditions, afforded X-ray structurally characterized heterobimetallic Cr^0^/Au^I^ ensemble [(OC)_5_Cr(μ-η^1^:η^1^-2-isocyano-1,3-diethoxycarbonyl-6-azulenylthiolate)AuPPh_3_] (**8**). Analysis of the ^13^C NMR chemical shifts for the [(NC)Cr(CO)_5_] core in a series of the related complexes [(OC)_5_Cr(2-isocyano-6-X-1,3-diethoxy-carbonylazulene)] (X = –N≡C, Br, H, SH, SCH_2_CH_2_CO_2_CH_2_CH_3_, SAuPPh_3_) unveiled remarkably consistent inverse-linear correlations *δ*(^13^CO_*trans*_) *vs. δ*(^13^CN) and *δ*(^13^CO_*cis*_) *vs. δ*(^13^CN) that appear to hold well beyond the above 2-isocyanoazulenic series to include complexes [(OC)_5_Cr(CNR)] containing strongly electron-withdrawing substituents R, such as CF_3_, CFClCF_2_Cl, C_2_F_3_, and C_6_F_5_. In addition to functioning as a sensitive ^13^C NMR handle, the essentially *C*_4v_-symmetric [(–NC)Cr(CO)_5_] moiety proved to be an informative, remote, *ν*_N≡C_/*ν*_C≡O_ infrared reporter in probing chemisorption of **7** on the Au(111) surface.

## Abstract 2

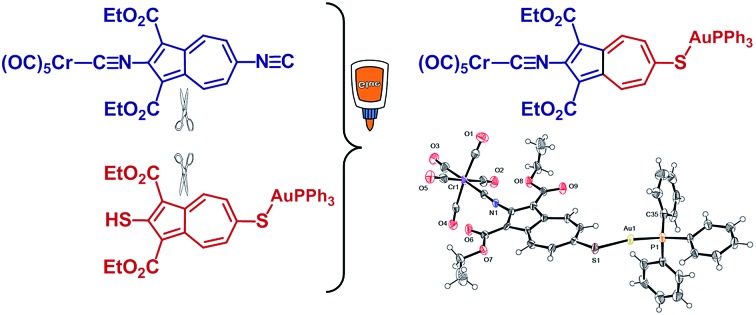
Azulene is a convenient platform for accessing heterobimetallic complexes and self-assembled monolayers of a π-linker with asymmetric junctions.

## Introduction

Mercapto (–SH) and isocyano (–N≡C) substituents are among particularly popular anchoring groups in coordination and surface chemistry as they are well-known to provide stable junctions at metal/organic interfaces.^1^–^3^ Even though dimercapto- and diisocyano-functionalized molecular linkers have long been attracting interest of theorists^4^–^9^ and experimentalists^10^–^15^ in the quest for efficient organoelectronic materials,^16^–^20^ species containing both –SH and –N≡C functionalities in the same molecule are not presently known and constitute a formidable synthetic challenge. Indeed, a mercapto group is incompatible with reaction conditions commonly employed to form an isocyano substituent,^21^ whereas free organic isocyanides are unlikely to tolerate chemical environments typically involved in the syntheses of mercaptans (thiols).^22^–^24^ In the context of targeting isocyanothiols for bridging metal-based electron reservoirs, a potentially straightforward strategy to circumvent the above dilemma would be to anchor either the –N≡C or the –SH terminus of such a hypothetical linker prior to forming and tethering its other end. There is only one related example in the literature, albeit not involving a mercapto group *per se* but rather its disulfide surrogate.^25^,^26^ In their elegant approach to covalently bind nickel clusters to a gold surface *via* the 4-isocyanophenylthiolate bridge, Kubiak and coworkers attached both –N≡C ends of otherwise non-isolable 1,2-bis(4-isocyanophenyl)disulfide to trinuclear nickel clusters in the μ_3_,η^1^ fashion.^25^ The resulting salt, [{Ni_3_(μ_3_-I)(μ_2_-dppm)_3_(μ_3_,η^1^-C≡NC_6_H_4_S–)}_2_]^2+^(I^–^)_2_ (dppm = bis(diphenylphosphino)methane), underwent homolysis of its S–S moiety upon exposure to a gold surface to give rectifying, presumably ionic, monolayer films.^26^

Earlier this year, Ratner and van Dyck proposed a new paradigm for the design of efficient molecular rectifiers that involved two π-conjugated units asymmetrically anchored to metallic electrodes and separated by a decoupling bridge.^27^ Their intriguing theoretical study suggested mercapto and cyano (–C≡N) junctions for accommodating the asymmetric anchoring on the premises that the –SH and –C≡N termini would facilitate alignments of a linker's HOMO (Highest Occupied Molecular Orbital) and LUMO (Lowest Unoccupied Molecular Orbital), respectively, through Fermi level pinning.^27^ We note that, from a practical standpoint, mercapto/isocyano asymmetric anchoring would be worth considering as well, given that the isocyano group offers a substantially more stable junction within a wider range of organometallic platforms compared to its isomeric cyano congener.^2^

**Fig. 1:**
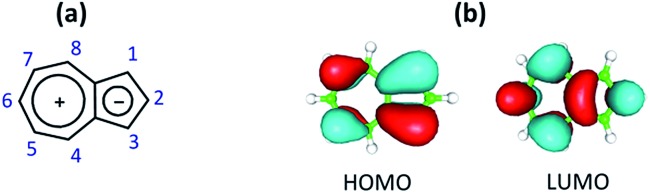
(a) Polar resonance form of azulene and C-atom numbering scheme of the azulenic scaffold; (b) Frontier molecular orbitals of azulene.

Herein, we introduce chemistry of the first, to the best of our knowledge, π-linker equipped with mercapto *and* isocyano anchoring groups. The linker's core is comprised of the non-alternant aromatic framework of azulene, a substitution-free molecular diode which has, among other unusual physico-chemical characteristics, complementary orbital density distributions within its Frontier molecular orbitals (Fig. 1).^28^

## Results and discussion

**Fig. 2:**
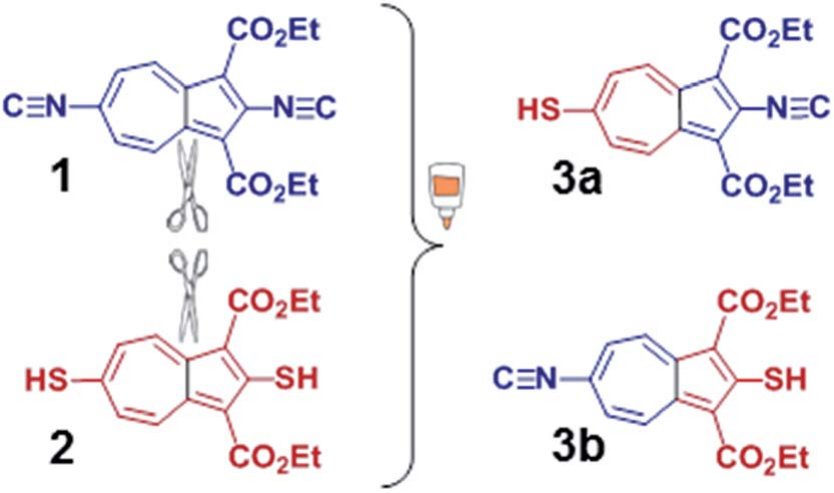
2,6-Diisocyano-1,3-diethoxycarbonylazulene (**1**),^32^ 2,6-dimercapto-1,3-diethoxy-carbonylazulene (**2**),^29^ and their hypothetical hybrids **3a** and **3b**.

**Fig. 3:**
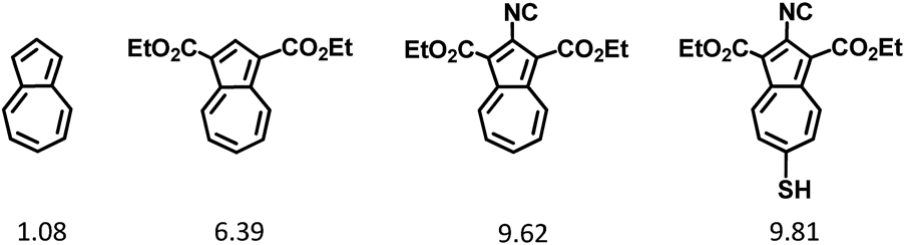
DFT-calculated electric dipole moments (in Debye) of azulene and its derivatives.

Recent synthetic breakthroughs in functionalization of the azulenic scaffold along its molecular axis have expanded the toolbox for developing low band-gap conducting and optoelectronic materials.^29^–^33^ The design of the title π-conjugated linker was influenced by and capitalized on our earlier studies involving 2,6-diisocyano- and 2,6-dimercapto-1,3-diethoxycarbonylazulenes, shown in Fig. 2 (compounds **1** and **2**, respectively). As illustrated in Fig. 2, one can envision pursuing two hybrids of **1** and **2**: 2-isocyano-6-mercapto-1,3-diethoxycarbonylazulene (**3a**) and 2-mercapto-6-isocyano-1,3-diethoxycarbonylazulene (**3b**). Among these two hybrids, **3a** is particularly interesting because each substituent in its structure reinforces the molecular dipole of the azulenic framework. In fact, our Density Functional Theory (DFT) calculations suggest that the dipole moment of the “parent” azulene molecule should increase nearly 10-fold upon incorporation of all substituents to form **3a** (Fig. 3).

**Scheme 1:**
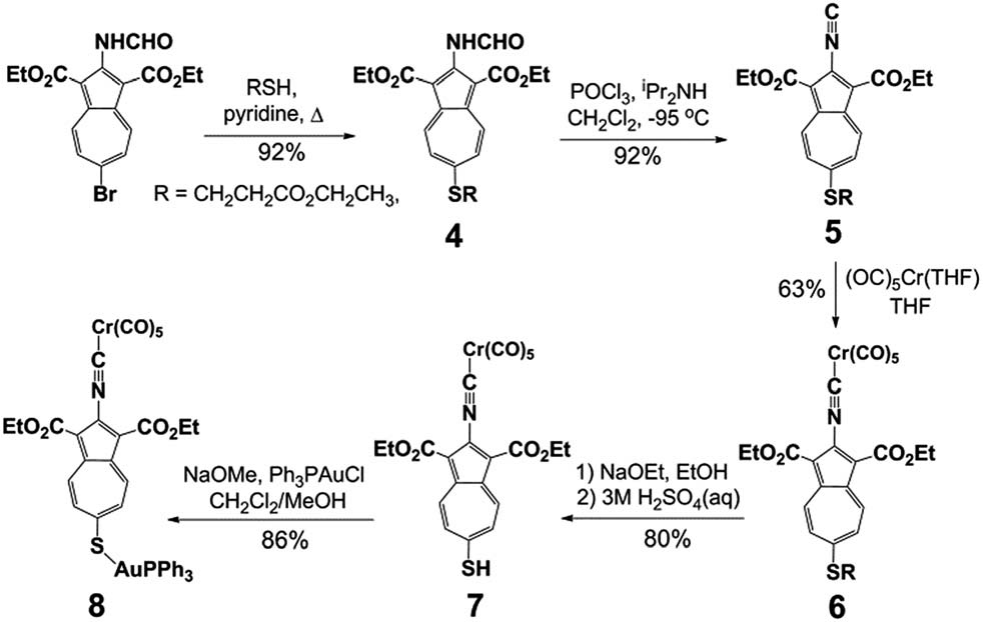
Synthesis and metalation of the 2-isocyano-6-mercaptoazulene motif.

Our synthetic approach to constructing and metalating **3a** is shown in Scheme 1. Treating pink 2-formamido-6-bromo-1,3-diethoxycarbonylazulene^32^,^34^ with ethyl 3-mercapto-propionate in refluxing pyridine afforded persimmon-coloured thioether **4** in a high yield. Dehydrating the 2-formamido group of **4** cleanly provided peach-red 2-isocyanoazulene derivative **5**. Unlike 1,2-bis(4-isocyanophenyl)disulphide (*vide supra*),^25^**5** is thermally and air-stable for practical purposes and can be stored under ambient conditions for at least a few weeks without spectroscopically (^1^H NMR, FTIR) detectable deterioration. Compound **5** reacted with Cr(CO)_5_(THF) *via* its 2-NC end to form orange Cr^0^ adduct **6**. No product featuring the thioether S → Cr(CO)_5_ interaction^35^ was documented in this reaction. The [(–NC)Cr(CO)_5_] moiety of **6** tolerated the basic environment and subsequent acidification of the reaction mixture used to convert **6** into auburn organometallic thiol **7**, which constitutes **3a** with its 2-NC terminus anchored to the 16-e^–^ [Cr(CO)_5_] fragment. Metalation of the 6-SH end of **7** with PPh_3_AuCl under basic conditions yielded orange-red crystals of heterobimetallic Cr^0^/Au^I^ complex **8** after a simple workup.

**Fig. 4:**
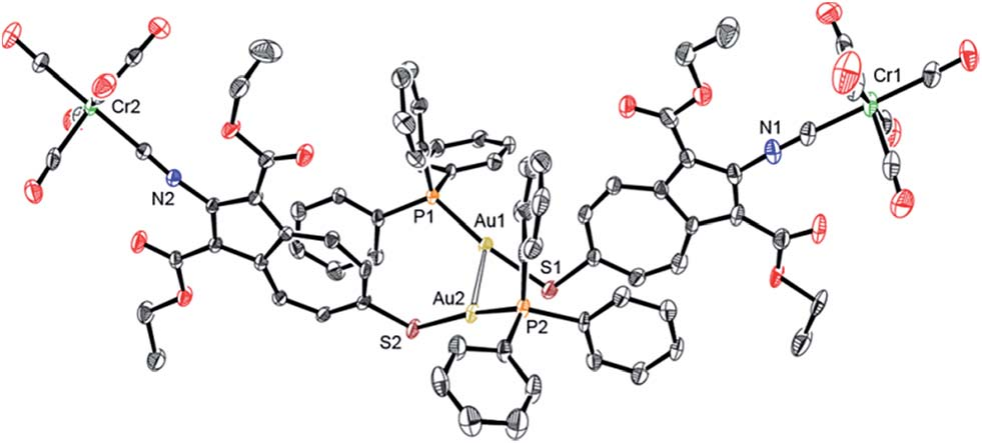
ORTEP diagram (50% thermal ellipsoids) of the asymmetric unit of **8**·¾CH_2_Cl_2_ emphasizing weak aurophilic interaction between two crystallographically independent molecules of **8**. The disordered CH_2_Cl_2_ molecules or crystallization (Fig. S4†) and H-atoms are omitted for clarity.

**Fig. 5:**
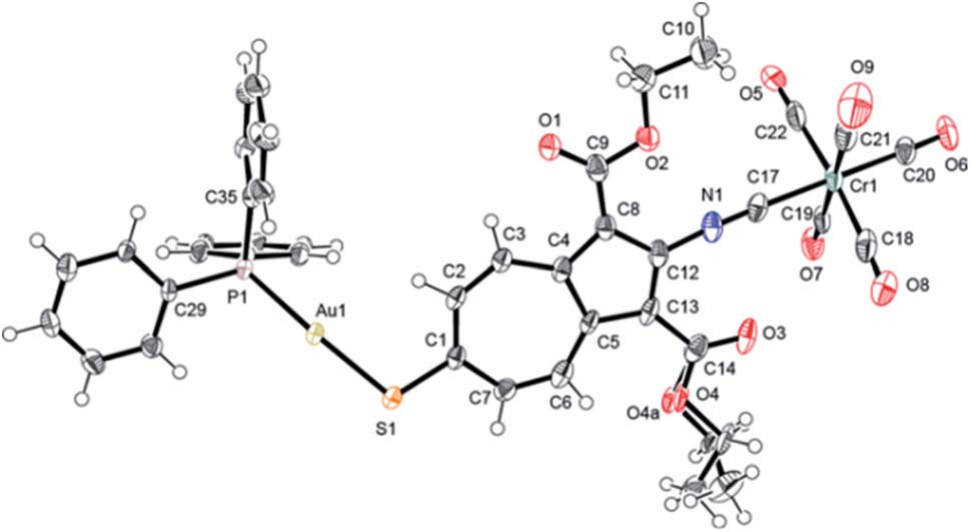
Molecular structure of one of the two crystallographically independent molecules of **8** (50% thermal ellipsoids). The OEt unit attached to C14 exhibits a positional disorder (Fig. S4†). Selected interatomic distances (Å) and angles (°): Au1–P1 2.268(1), Au1–S1 2.318(1), S1–C1 1.744(5), Cr1–C17 1.960(6), Cr1–C18 1.901(8), Cr1–C19 1.900(6), Cr1–C20 1.891(6), Cr1–C21 1.901(7), Cr1–C22 1.891(7), C17–N1 1.155(7), C18–O8 1.155(8), C19–O7 1.141(6), C20–O6 1.142(6), C21–O9 1.146(7), C22–O5 1.147(7), P1–Au1–S1 165.82(5), Au1–S1–C1 108.1(2), C12–N1–C17 172.6(6), Cr1–C17–N1 175.3(5).

**Fig. 6:**
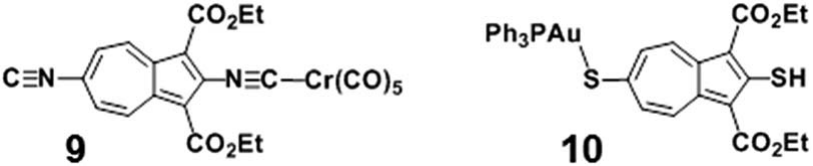
Previously X-ray structurally characterized mononuclear Cr^0^ and Au^I^ complexes **9** (ref. 32) and **10** (ref. 29).

The solid-state structure of **8**·¾CH_2_Cl_2_ features two very similar but crystallographically independent molecules of **8** in the asymmetric unit that are linked together *via* a weak Au···Au interaction^36^ of 3.2102(4) Å (Fig. 4, 5, S3 and S4†). The partially positively charged 7-membered ring of the highly polarizable azulenic moiety in each of these molecules of **8** undergoes donor–acceptor face-centred stacking^37^ with a Ph-ring of the other molecule's PPh_3_ ligand giving the intercentroid distances^38^ of 3.65 and 3.76 Å. Heterobimetallic complex **8** may be viewed as a hybrid of our X-ray structurally characterized mononuclear Cr^0^ and Au^I^ adducts of **1** and **2**, respectively, depicted in Fig. 6 (complexes **9** (ref. 32) and **10** (ref. 29)). While the S–Au–P unit in **10** is practically linear (*ca.* 177.4°),^29^,^39^ bending of the S–Au–P angle (*ca.* 166.5°) in **8** is undoubtedly a consequence of the Au···Au bonding reinforced further by the “aromatic donor-acceptor interactions”.^37^ The above structural perturbations do not significantly affect the Au–S–C angle in **8** compared to that in **10**, which are *ca.* 107.8° 105.0°, respectively. Notably, the solid state structure of **10** exhibits neither aurophilic nor aromatic stacking interactions akin to those observed for **8**.^29^

The metric parameters for the octahedral [(–NC)Cr(CO)_5_] core in **8** are quite similar to those observed for **9** (ref. 32) and many other complexes (ArylNC)Cr(CO)_5_.^39^ Comparison of the Cr–CN and C≡N bond distances^40^ for **8** and **9** (Table 1) may hint that the 2-isocyanoazulene ligand in **8** has a somewhat higher σ-donor/π-acceptor ratio than that in **9**, thereby reflecting the difference in electron-donating/withdrawing characteristics of –SAuPPh_3_*versus* –N≡C groups at position 6 of the azulenic scaffold. However, this suggestion should be taken *cum grano salis* as such subtle variations in *d*(Cr–CN) and *d*(C≡N) are statistically ambiguous, especially under the 3σ criterion. More drastic changes in the electronic nature of the isocyanide ligand's substituent do lead to significant alterations in the Cr–CN and C≡N bond lengths in (RNC)Cr(CO)_5_ as illustrated in Table 1 for R = ^*t*^Bu (ref. 42) and FC≡CF_2_.^43^

**Table 1:** Selected bond distances and angles for **8**, **9**, and (RNC)Cr(CO)_5_ (R = ^*t*^Bu, C_2_F_3_)

	*d*(Cr–CN), Å	*d*(C≡N), Å	∠(C–N–C), °
(^*t*^BuNC)Cr(CO)_5_^*a*^	2.016(2)	1.150(2)	177.9(2)
**8** ^*b*^	1.960(6), 1.969(5)	1.155(7), 1.158(6)	172.6(6), 173.3(5)
**9** ^*c*^	1.953(4)	1.166(4)	167.5(3)
(F_3_C_2_NC)Cr(CO)_5_^*d*^	1.909(2)	1.162(2)	173.6(2)

^*a*^ Ref. 41.

^*b*^Data for two crystallographically unique molecules.

^*c*^ Ref. 32.

^*d*^ Ref. 43.

**Fig. 7:**
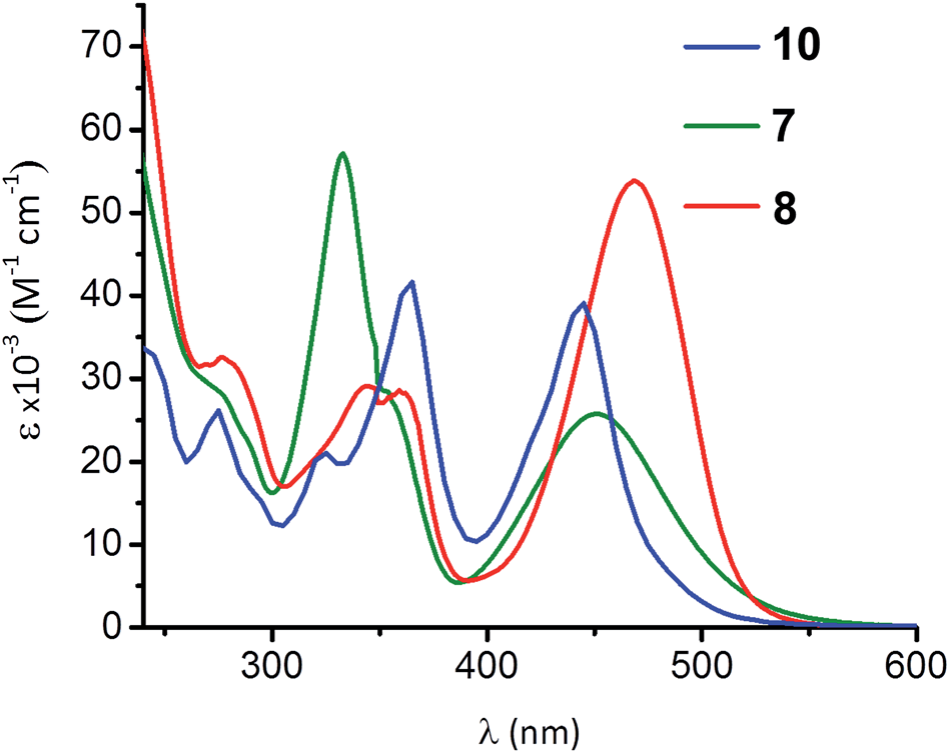
UV-vis spectra of **7**, **8**, and **10** (ref. 29) in CH_2_Cl_2_ at 25 °C.

**Fig. 8:**
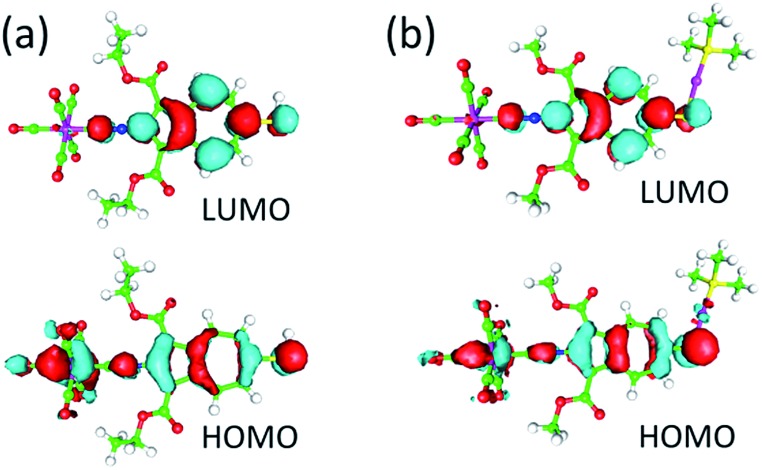
DFT-calculated Frontier MO's of (a) **7** and (b) **8a**, a truncated model of **8**.

Compounds **4–8** are highly coloured substances. The lowest energy electronic absorption band for **5** occurs at 484 nm (*ε* = 1.55 × 10^3^ M^–1^ cm^–1^) and is 259 cm^–1^ red-shifted compared to the S0 → S1 transition documented for **4** (Fig. S1†). This red shift arises from the greater electron-withdrawing influence of the 2-isocyano group in **5***versus* the 2-formamido group in **4** on the energy of the azulenic scaffold's LUMO (Fig. 1b).^31^,^44^ The UV-vis spectra of **6** and **7** are nearly identical and feature very intense absorption bands at 454 (*ε* = 3.1 × 10^4^ M^–1^ cm^–1^) and 452 (*ε* = 2.6 × 10^4^ M^–1^ cm^–1^), respectively, that have a substantial contribution from the *d*π(Cr) → pπ*(CNAzulenyl) charge transfer (Fig. 7 and S1†). Our time-dependent DFT (TD-DFT) calculations for **7** suggest that the transition at 452 nm (TD-DFT: 416 nm) has 85% HOMO → LUMO character (Fig. 8a). Upon metalation of **7** to form **8**, this band not only red-shifts to 469 nm (TD-DFT: 463 nm for **8a**, the truncated model of **8** featuring OMe and PMe_3_ groups instead of OEt and PPh_3_, respectively, Fig. 8b) but also more than doubles in intensity (*ε* = 5.4 × 10^4^ M^–1^ cm^–1^). This intensity gain is due to the addition of the n(S) → pπ*(CNAzulenyl) character to the HOMO → LUMO transition observed for **8** (*cf.* the 445 nm band for **10** in Fig. 7).^29^ As in the case of **9** and **10**,^29^,^32^ the LUMOs of **7** and **8a** constitute the π*-system of the azulenic moiety with contributions from both anchoring groups while their HOMOs involve the entire 2-isocyano-6-azulenylthiolate motif (Fig. 8).

Whilst considering ^13^C NMR signatures of the [(NC)Cr(CO)_5_] core in **6**, **7**, **8**, and **9**, we noticed that they were predictably sensitive to the nature of the substituent at position 6 of the azulenic scaffold. To further validate this initial observation, we expanded the above family of four related complexes [(OC)_5_Cr(2-isocyano-6-X-1,3-diethoxycarbonylazulene)] (X = SCH_2_CH_2_CO_2_CH_2_CH_3_, SH, SAuPPh_3_, N≡C) to include species with X = H (**11**) and Br (**12**). The top six rows in Table 2 contain ^13^C NMR data pertaining to the [(NC)Cr(CO)_5_] moiety in this series of six 2-isocyanoazulenic adducts. All of these ^13^C NMR measurements were performed for samples dissolved in CDCl_3_.

**Table 2:** ^13^C NMR data for the [Cr(CO)_5_(CN)] moiety in complexes (RNC)Cr(CO)_5_^*a*^

Compound	*δ*(^13^CN)	*δ*(^13^CO_*trans*_)	*δ*(^13^CO_*cis*_)
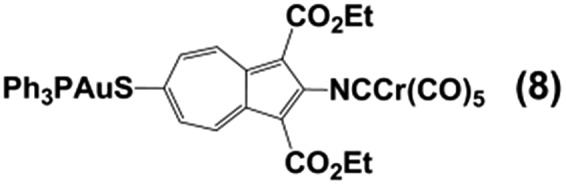	178.55	217.26	214.91
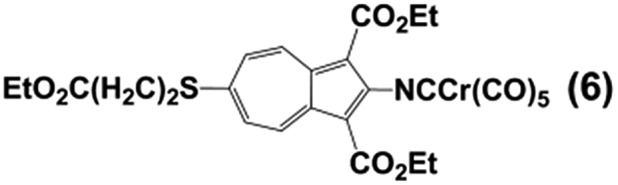	181.72	216.85	214.68
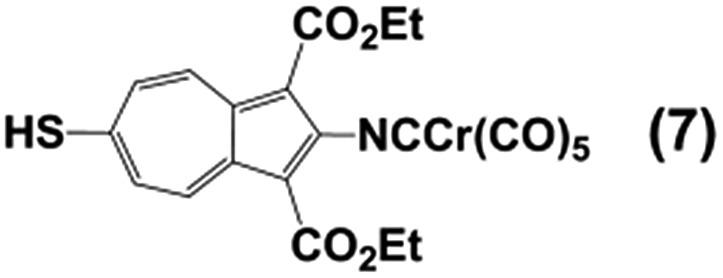	182.27	216.77	214.65
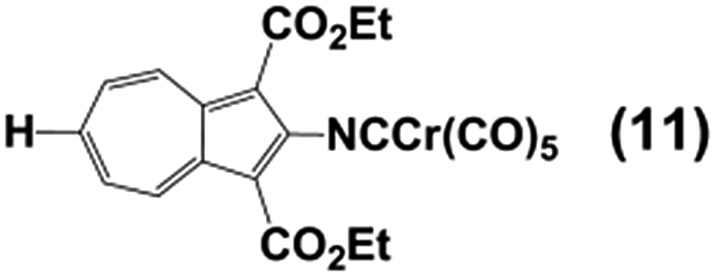	183.36	216.69	214.60
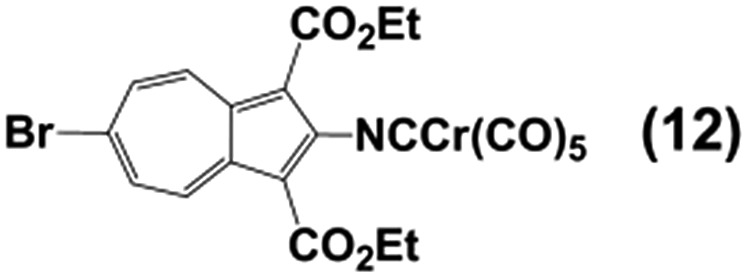	184.42	216.52	214.47
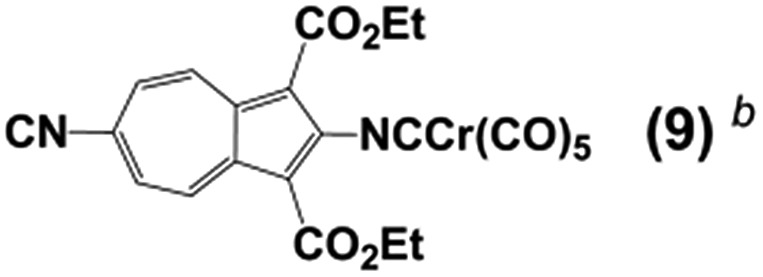	186.6	216.3	214.4
F_5_C_6_–NCCr(CO)_5_^*c*^	193.8	214.6	213.3
F_2_C={F}C–NCCr(CO)_5_^*d*^	199.3	214.2	213.0
ClF_2_C{ClF}C–NCCr(CO)_5_^*d*^	208.2	212.0	212.0
F_3_C–NCCr(CO)_5_^*e*^	211.1	211.5	211.7

^*a*^All spectra were recorded in CDCl_3_.

^*b*^ Ref. 32.

^*c*^ Ref. 47.

^*d*^ Ref. 43.

^*e*^ Ref. 48.

From Table 2 it is evident that as the *net* electron-releasing ability of X decreases (SAuPPh_3_ > SCH_2_CH_2_CO_2_CH_2_CH_3_ > SH > H > Br > N≡C), the *δ*(^13^CN) value for the isocyano carbon resonance increases in the range spanning *ca.* 8 ppm, thereby signifying gradual drop in the σ-donor/π-acceptor ratio of the 2-isocyano-6-X-azulene ligand. Concomitantly, both *δ*(^13^CO_*trans*_) and *δ*(^13^CO_*cis*_) values decrease, albeit in tighter chemical shift ranges (∼1.0 and ∼0.5 ppm, respectively), indicating reduction in the electron richness of the Cr-centre. Even though the ^13^C chemical shifts of terminal CO and CNR ligands in low-valent complexes are influenced considerably by the paramagnetic shielding term, σ^*para*^, which reflects the degree of π-backbonding,^40^,^45^,^46^ it is more appropriate to interpret Δ*δ*(^13^CN) and Δ*δ*(^13^CO) as a combined σ-donor/π-acceptor effect.

**Fig. 9:**
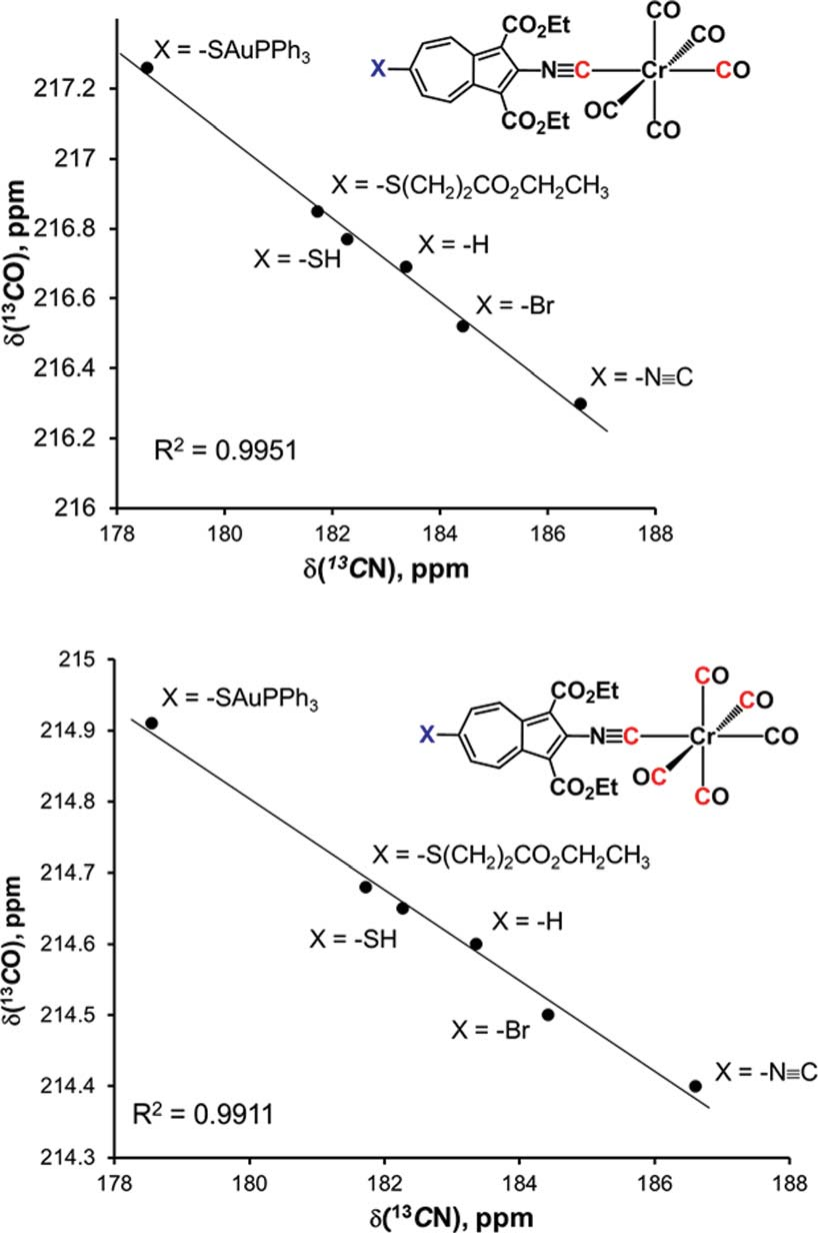
(a) Plot of *δ*(^13^CO_*trans*_) *vs. δ*(^13^CN) chemical shifts (in CDCl_3_) in the ^13^C NMR spectra of **6**, **7**, **8**, **9**, **11**, and **12**; (b) plot of *δ*(^13^CO_*cis*_) *vs. δ*(^13^CN) chemical shifts (in CDCl_3_) in the ^13^C NMR spectra of **6**, **7**, **8**, **9**, **11**, and **12**.

**Fig. 10:**
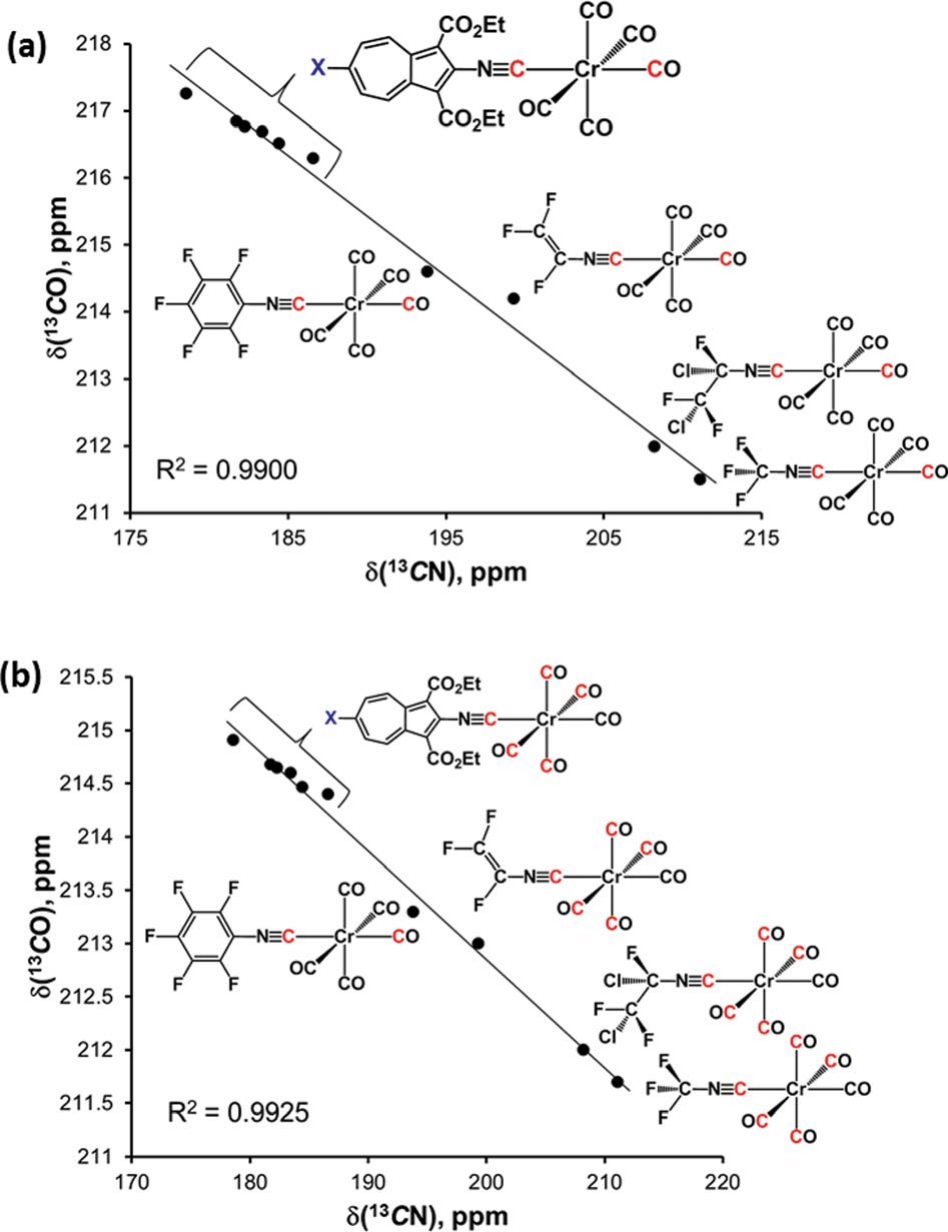
(a) Plot of *δ*(^13^CO_*trans*_) *vs. δ*(^13^CN) chemical shifts (in CDCl_3_) in the ^13^C NMR spectra of all compounds from Table 2; (b) plot of *δ*(^13^CO_*cis*_) *vs. δ*(^13^CN) chemical shifts (in CDCl_3_) in the ^13^C NMR spectra of all compounds from Table 2.

Closer examination of the ^13^C NMR data in the top six rows of Table 2 unveiled remarkably consistent inverse-linear relationships *δ*(^13^CO_*trans*_) *vs. δ*(^13^CN) and *δ*(^13^CO_*cis*_) *vs. δ*(^13^CN), as illustrated in Fig. 9. This figure also confirms that remote modulation the Cr-centre's electron richness mediated by the 2,6-azulenic framework affects the *trans*-CO ligand to a greater extent than the *cis*-CO's of the [(NC)Cr(CO)_5_] moiety. Would the trends depicted in Fig. 9 hold beyond the 2-isocyanoazulenic series? To address this question, we considered (RNC)Cr(CO)_5_ species containing strongly electron-withdrawing substituents R, for which ^13^C NMR data acquired in *the same solvent* (CDCl_3_) were available (bottom four rows in Table 2). The expanded *δ*(^13^CO_*trans*_) *vs. δ*(^13^CN) and *δ*(^13^CO_*cis*_) *vs. δ*(^13^CN) plots that, in addition to the 2-isocyanoazulenic complexes, include (OC)_5_Cr(CNR) with R = C_6_F_5_,^47^ C_2_F_3_,^43^ CFClCF_2_Cl,^43^ and CF_3_ (ref. 48) are shown in Fig. 10, which again demonstrates excellent inverse-linear correlations now spanning substantially wider Δ*δ*(^13^CN) and Δ*δ*(^13^CO) windows.

**Fig. 11:**
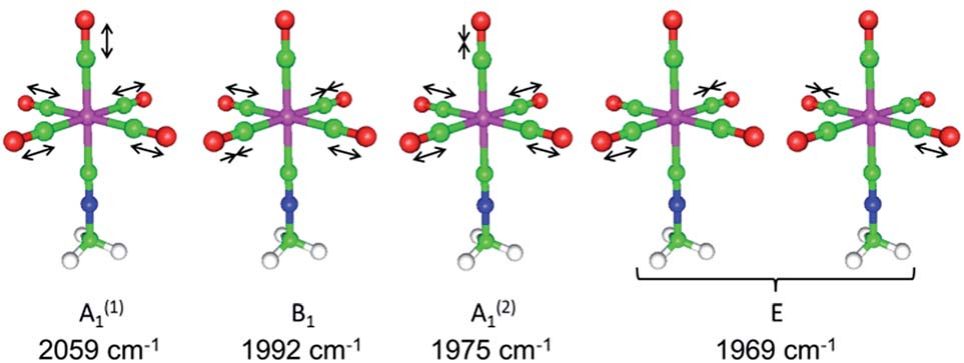
DFT-calculated *ν*_CO_ vibrational profile for (MeNC)Cr(CO)_5_ in the gas phase.^55^,^56^

The above *δ*(^13^CO)/*δ*(^13^CN) NMR analysis serves as a convenient tool for quantifying even subtle electronic influence of a CNR ligand's substituent R. In this regard, it offers a simple alternative to the well-established method involving correlation the carbonyl ^13^C chemical shifts with the corresponding CO force constants (*k*_CO_) for complexes (RNC)Cr(CO)_5_.^40^,^49^,^50^ Unfortunately, changes in *k*_CO_ due to mild electronic perturbations of the R group are often not clearly discernible.^40^,^51^ Determining the values of *k*_CO_'s under the *C*_4v_ symmetry for complexes (RNC)_5_Cr(CO)_5_ using the Cotton–Kraihanzel (C–K) approximation^52^ is a straightforward but somewhat tedious task that carries fundamental limitations^52^–^54^ and relies on the availability of the complete *ν*_C≡O_ vibrational profile^52^ (*Γ*_*ν*CO_ = 2A_1_ + B_1_ + E, *e.g.*, Fig. 11 (ref. 55 and 56) and Table S17†). In the IR spectra of LM(CO)_5_ species, the lower energy *ν*_C≡O_(A_1_) band is often obscured by the intense *ν*_C≡O_(E) band,^40^,^51^–^54^ which compromises the accuracy of experimental determination of this *ν*_C≡O_(A_1_) value (*vide infra*).

Similar to the trend in *δ*(^13^CN) for the (RNC)Cr(CO)_5_ adducts in Table 2, the ^13^C NMR resonance for the terminal C-atom in the available uncoordinated 2-isocyanoazulenes moves upfield upon increasing electron-donating power of the substituent X at the azulenic 6-position (*δ* = 179.9,^32^ 178.0, 177.5, 176.3 ppm in CDCl_3_ for X = ≡C, Br, H, SCH_2_CH_2_CO_2_CH_2_CH_3_, respectively). Yet, the *ν*_N≡C_ stretching frequency for these free 2-isocyano-azulenes (2126 ± 1 cm^–1^ in CH_2_Cl_2_) is insensitive to the nature of the group X. However, upon proceeding from **8** to (**6**, **7**, **11**) to **12** to **9**, the *ν*_N≡C_ band undergoes a small red shift (Table 3), thereby suggesting decrease in the σ-donor/π-acid ratio of the isocyanide ligand, especially when **8** is compared to **9** and **12**.

**Table 3:** IR signatures of the [(NC)Cr(CO)_5_] core in **6**, **7**, **8**, **9**, **11**, and **12** (in CH_2_Cl_2_)

	*ν*_NC_(A_1_), cm^–1^	*ν*_CO_(A_1_^(1)^), cm^–1^	*ν*_CO_(B_1_), cm^–1^	*ν*_CO_(A_1_^(2)^ + E), cm^–1^
**8**	2144	2054	2003	1957
**6**	2140	2050	2000	1958
**7**	2140	2049	2000	1958
**11**	2140	2049	2001	1959
**12**	2137	2047	2002	1960
**9** ^*a*^	2135	2043	2002	1962

^*a*^ Ref. 32.

**Fig. 12:**
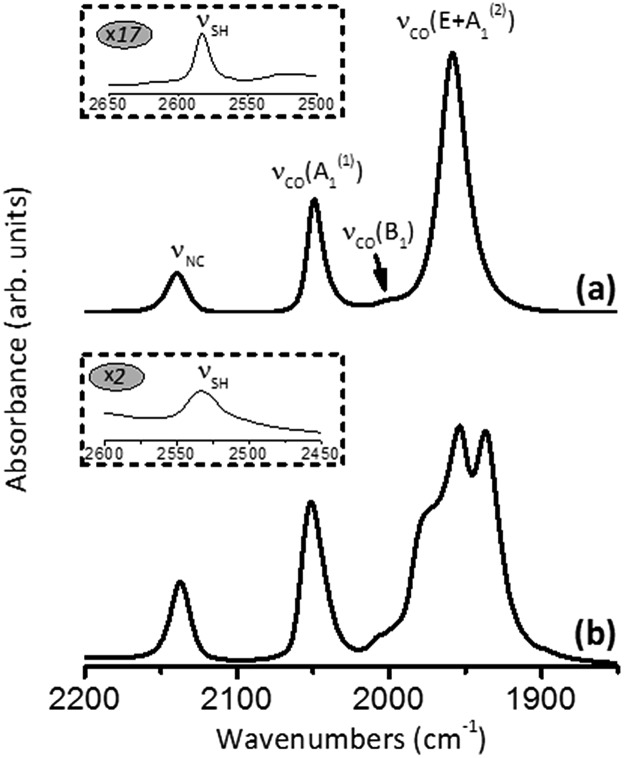
FTIR spectra of **7** in (a) CH_2_Cl_2_ and (b) KBr.

Fig. 12a shows the FTIR spectrum of thiol **7** in CH_2_Cl_2_. In addition to the characteristic *ν*_S–H_ and *ν*_N≡C_ bands at 2583 and 2140 cm^–1^, respectively, it features a typical pattern in the *ν*_C≡O_ stretching region for a LM(CO)_5_ species.^57^ The band at 2049 cm^–1^ corresponds to the *ν*_C≡O_ mode A_1_^(1)^ where all five CO ligands vibrate in-phase (*cf.*Fig. 11). The very weak band at 2000 cm^–1^ is due to the *ν*_C≡O_ vibration of B_1_-symmetry, which is IR-forbidden under the strict *C*_4v_ symmetry but gains slight intensity because of minor deviations of the structure from the idealized *C*_4v_ geometry. The intense *ν*_C≡O_ band at 1958 cm^–1^ chiefly represents the doubly degenerate vibration of E-symmetry. This *ν*_C≡O_(E) band obscures the remaining IR-active *ν*_C≡O_ mode A_1_^(2)^. Interestingly, perturbations of the local *C*_4v_ symmetry in **7** through crystal packing interactions in the solid state are sufficient to split the E-mode into two separate *ν*_C≡O_ peaks while unmasking the original A_1_^(2)^ mode (Fig. 12b).

**Fig. 13:**
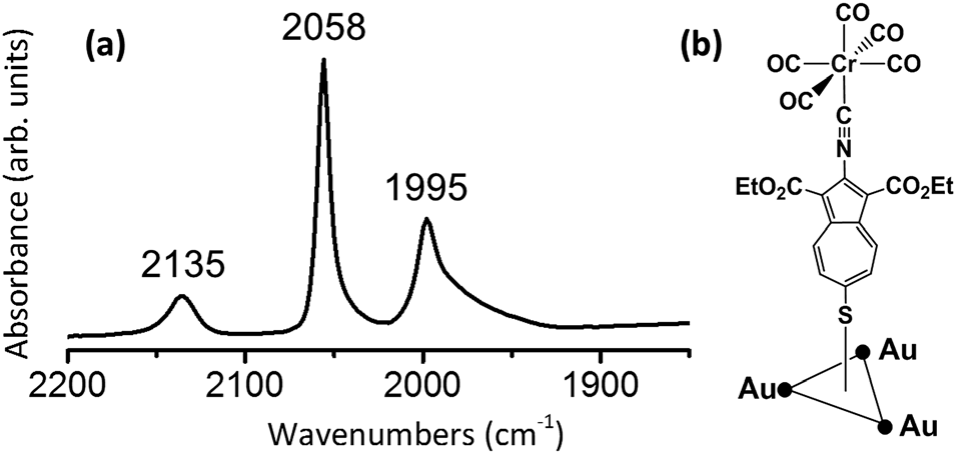
(a) RAIR spectra of **7** adsorbed on Au(111); (b) the “hollow-linear” coordination mode of **7** adsorbed on Au(111).

Exposing *ca.* 1 × 1 cm^2^ gold substrates to a 2 mM solution of **7** in CHCl_3_ without protection from air and ambient lighting reproducibly afforded self-assembled monolayer (SAM) films of **7** on the Au(111) surface. This chemisorption process is presumably accompanied by formation of the thiolate junction and the release of H_2_.^8^,^17^,^58^ The reflection absorption infrared (RAIR) spectrum of the SAM of **7** on Au(111) is shown in Fig. 13a. In addition to the *ν*_N≡C_ absorption at 2135 cm^–1^, it features two *ν*_C≡O_ bands. The *ν*_C≡O_ region in this RAIR spectrum, however, is quite different from that in Fig. 11a in terms of peak intensities and energies. The lowest energy intense *ν*_C≡O_ band in the solution IR spectrum of **7**, which is primarily attributed to the *ν*_C≡O_ mode of E symmetry, practically vanishes upon the SAM formation, while simultaneously uncovering the hidden A_1_^(2)^ band of much lower intensity. This observation implies approximately parallel orientation of the *cis*-CO ligands with respect to the gold surface. Indeed, surface IR selection rules^59^ dictate that only vibrations contributing to dipole changes perpendicular to the surface are IR-active. Consequently, any vibrations occurring nearly parallel to the surface would have low IR intensity. Given that the C–N–C unit in **7** is expected to be essentially linear, the appearance of the RAIR spectrum in Fig. 13a suggests upright orientation (*i.e.*, straight C–S–Au_surface_ angle) of the molecules in the SAMs of **7**.

The “hollow-linear” coordination of organic thiolates in their SAMs on Au(111), akin to that depicted in Fig. 13b, has been predicted to accommodate the strongest S–Au interaction and induce S → Au(111) charge transfer *via* S(3p)–Au π-bonding.^60^,^61^ In the context of the chemistry presented herein, this means that the gold surface would effectively function as an electron-withdrawing “substituent”, thus, enhancing π-acidity of the 2-isocyanoazulene ligand and, in turn, decreasing electron richness of the [Cr(CO)_5_] unit. The A_1_^(1)^ and A_1_^(2)^*ν*_C≡O_ bands at 2058 and 1995 cm^–1^ in the RAIR spectrum in Fig. 11 both exhibit significant blue shifts compared to the corresponding *ν*_C≡O_ peaks in the solution FTIR spectrum of **7** (2049 and 1958 cm^–1^, respectively, Fig. 10a). The magnitudes of these shifts appear to be too high, especially in the case of the A_1_^(2)^ mode, to be attributed solely to differences in intermolecular interactions within the SAM *vs.* solution of **7**. The larger change in energy of the *ν*_C≡O_ A_1_^(2)^ mode compared to that of the A_1_^(1)^ mode upon chemisorption of **7** stems from the greater contribution of the *trans*-CO stretch to the former.^62^

The tilt angle of the aromatic moiety in SAMs of benzenoid mercaptoarenes on Au(111) can be highly variable.^17^ We have recently shown that 2-mercaptoazulene and several of its derivatives form monolayer films on Au(111) with approximately upright assembly of the azulenylthiolate constituents.^63^ Our optical ellipsometry measurements on multiple SAM samples of **7** provided consistent SAM thickness values that nicely corroborate the monolayer nature of these films and upright orientation of the molecules on the gold surface (Table 4). In terms of their composition, the SAMs of **7** and **9** on Au(111) differ only in the surface anchoring group (thiolate *vs.* isocyanide) and appear to exhibit essentially identical thicknesses.^64^ Notably, neither RAIR spectroscopic nor ellipsometric data collected for the SAMs of **7** on Au(111) would be consistent with the “on-top-bent”^59^ or any other adsorption models of **7** invoking a bent C–S–Au_surface_ geometry. The ellipsometric measurements on SAM films formed from our recently reported^29^ 6-mercapto-1,3-diethoxycarbonyl-azulene and 6-mercapto-2-chloro-1,3-diethoxycarbonylazulene also corroborate that these 6-mercaptoazulenes self-assemble on Au(111) surfaces in the upright fashion (Table 4).

**Table 4:** Observed ellipsometric (*D*_obs_) and calculated (*D*_calc_) film thicknesses (in Å) of the SAMs of **7**, 6-mercapto-1,3-diethoxycarbonylazulene, and 6-mercapto-2-chloro-1,3-diethoxycarbonylazulene

Mercaptoazulene derivative	*D*_obs_ ^*a*^	*D*_calc_ ^*b*^
**7**	18.3 ± 2.7	17.1
6-Mercapto-1,3-diethoxycarbonylazulene	12.8 ± 1.9	13.3
6-Mercapto-2-chloro-1,3-diethoxycarbonylazulene	14.6 ± 1.9	13.3

^*a*^Average of five measurements at different spots on multiple SAM samples.

^*b*^Calculated from the X-ray structural data for **8**, 6-mercapto-1,3-diethoxycarbonylazulene (ref. 29), and 6-mercapto-2-chloro-1,3-diethoxy-carbonylazulene (ref. 29), as well as by assuming straight C–S–Au_surface_ angle and the Au(111)–S distance of 2.45 Å (ref. 58).

## Conclusions

The asymmetric nonbenzenoid aromatic framework of azulene proved to be a convenient platform for accessing the first π-linker terminated with both mercapto and isocyano junction moieties. Anchoring the 2-isocyano end of this linker was an important prerequisite to successfully installing its 6-mercapto terminus. The ^13^C NMR signatures of the octahedral [(–NC)Cr(CO)_5_] core in related complexes **6**, **7**, **8**, **9**, **11**, and **12** provided a sensitive spectroscopic handle for tuning electron richness of the Cr^0^-centre through mediation by the 2,6-azulenic framework. Moreover, the remarkably consistent inverse-linear trends *δ*(^13^CO_*trans*_)/*δ*(^13^CN) and *δ*(^13^CO_*cis*_)/*δ*(^13^CN) for a wide spectrum of complexes (RNC)Cr(CO)_5_ offer a simple and more accurate alternative to the *δ*(^13^CO)/*k*_CO_ strategy in quantifying electronic influence of the substituent R in isocyanide ligands. This ^13^C NMR approach utilizes feedback from the entire [(–NC)Cr(CO)_5_] unit rather than focusing on the [Cr(CO)_5_] fragment in the *δ*(^13^CO)/*k*_CO_ method. In addition, the *C*_4v_-symmetric [(–CN)Cr(CO)_5_] moiety served as a distinctly informative *ν*_N≡C_/*ν*_C≡O_ infrared reporter for probing self-assembly of the 6-mercaptoazulenic motif on the Au(111) surface. We hope that the chemistry of the 2-isocyano-6-mercaptoazulenic platform introduced herein will facilitate further development and experimental validation of the emerging concept of asymmetric anchoring relevant to the design of organic electronics materials. Efforts to access and isolate completely free (*i.e.*, unmetalated) **3a** are currently in progress.

## Supplementary Material

Supplementary information

Crystal structure data
