# Supplementary material for: First π-linker featuring mercapto and isocyano anchoring groups within the same molecule: synthesis, heterobimetallic complexation and self-assembly on Au(111)
Source: Chem Sci. 2015 Nov 20;7(2):1422–9. doi: 10.1039/c5sc04017e (PMC4748958; doi:10.1039/c5sc04017e)
Supplement: Supplementary file 1 [file SC-007-C5SC04017E-s001.pdf]

Electronic Supplementary Material (ESI) for Chemical Science.
This journal is © The Royal Society of Chemistry 2015

Submitted to *Chemical Science*

Supplementary Information

for

First π-linker featuring mercapto and isocyano junctions within the same molecule: Synthesis, heterobimetallic complexation and self-assembly on Au(111)

Jason C. Applegate,[a] Monisola K. Okeowo,[a] Nathan R. Erickson,[a] Brad M. Neal,[a] Cindy L. Berrie*,[a] Nikolay N. Gerasimchuk*[b] and Mikhail V. Barybin*[a]

[a] *Department of Chemistry, The University of Kansas, 1251 Wescoe Hall Drive, Lawrence, KS 66045, USA*

[b] *Department of Chemistry, Missouri State University, 901 S. National Ave., Springfield, MO 65897, USA*

*To whom correspondence should be addressed:

E-mail: mbarybin@ku.edu, cberrie@ku.edu, NNGerasimchuk@MissouriState.edu
Fax: +1-785-864-5396; Tel: +1-785-864-4106

TABLE OF CONTENTS

A. Synthetic procedures and characterization **S-4**

A1. General procedures, starting materials and equipment S-4
A2. Synthesis of compound **4** S-4
A3. Synthesis of compound **5** S-5
A4. Synthesis of compound **6** S-5
A5. Synthesis of compound **7** S-6
A6. Synthesis of compound **8** S-6
A7. Synthesis of compound **11** S-7
A8. Synthesis of compound **12** S-7
Figure S1. UV-Vis spectra of **4**, **5**, and **6** in CH_2Cl_2 at 25 °C S-8

B. X-ray structure determination for 8·$^3/_4CH_2Cl_2$ **S-9**

B1. Experimental S-9
Table S1. Crystal data and structure refinement for **8**·$^3/_4CH_2Cl_2$ S-10
Table S2. Atomic coordinates and isotropic displacement parameters for **8**·$^3/_4CH_2Cl_2$ S-11
Table S3. Bond lengths [Å] for **8**·$^3/_4CH_2Cl_2$ S-15
Table S4. Bond angles [°] for **8**·$^3/_4CH_2Cl_2$ S-18
Table S5. Torsion angles [°] for **8**·$^3/_4CH_2Cl_2$ S-23
Figure S2. Photographs of the actual crystal of **8**·$^3/_4CH_2Cl_2$ used in the X-ray diffraction study S-25
Figure S3. ORTEP diagram of the asymmetric unit of **8**·$^3/_4CH_2Cl_2$ S-26
Figure S4. ORTEP diagram of one of the two crystallographically independent molecules of **8** in the asymmetric unit S-26
Figure S5. Detailed ORTEP drawings of the models used to resolve disordered ester groups in **8**·$^3/_4CH_2Cl_2$. S-27
Figure S6. ORTEP drawing of the disordered CH_2Cl_2 molecule of crystallization in **8**·$^3/_4CH_2Cl_2$ S-27

C. Surface Studies **S-28**

C1. Self-assembled monolayer films of **7** on the Au(111) surface S-28
C2. Optical ellipsometry S-28
C3. Surface IR measurements S-28

D. DFT Calculations **S-29**

D1. Experimental S-29
Table S6. Cartesian coordinates (Å) for the optimized structure of **7** S-30
Table S7. Cartesian coordinates (Å) for the optimized structure of **7** with implicit CH_2Cl_2 solvent S-32
Table S8. Energies and the corresponding oscillator strengths of the first 100 transitions from the TD-DFT calculations on **7** in CH_2Cl_2 S-34
Table S9. TD-DFT-calculated optical transitions for the lower energy portion of the electronic excitation spectrum of **7** S-37

Table S10. Cartesian coordinates (Å) for the optimized structure of **8a** S-38
Table S11. Energies and the corresponding oscillator strengths of the first 100 transitions from the TD-DFT calculations on **8a** S-40
Table S12. TD-DFT-calculated optical transitions for the lower energy portion of the calculated electronic excitation spectrum of **8a** S-43
Table S13. Cartesian coordinates (Å) for the optimized structure of azulene S-45
Table S14. Cartesian coordinates (Å) for the optimized structure of 1,3-diethoxycarbonylazulene S-46
Table S15. Cartesian coordinates (Å) for the optimized structure of 2-isocyano-1,3-diethoxycarbonylazulene S-47
Table S16. Cartesian coordinates (Å) for the optimized structure of 2-isocyano-6-mercapto-1,3-diethoxycarboylazulene (**3a**) S-48
Table S17. DFT-calculated (gas phase) and experimentally determined (IR, in *n*-hexane) ν_{NC} and ν_{CO} vibrational profile for C_{4v}-symmetric $(MeNC)Cr(CO)_5$ S-49

E. References **S-50**

A. SYNTHETIC PROCEDURES AND CHARACTERIZATION

A1. General procedures, starting materials and equipment. Unless specified otherwise, all operations were performed under an atmosphere of 99.5% argon purified by passage through columns of activated BASF catalyst and molecular sieves. All connections involving the gas purification systems were made of glass, metal, or other materials impermeable to air. In air-free procedures, solutions were transferred via stainless steel needles (cannulas). Standard Schlenk techniques were employed with a double manifold vacuum line. Both CH_2Cl_2 and CD_2Cl_2 were distilled over CaH_2, whereas $CHCl_3$ and $CDCl_3$ were distilled over P_2O_5. Tetrahydrofuran (THF) was distilled from Na/benzophenone. Pentane was distilled from Na/benzophenone dissolved in a minimum amount of diglyme. Pyridine was distilled over freshly cut sodium metal. Methanol and ethanol were distilled over Mg turnings. Other solvents were used as received from commercial sources.

Infrared spectra were recorded on a PerkinElmer Spectrum 100 FTIR spectrometer with liquid samples sealed in 0.1 mm NaCl cells or solid samples as KBr pellets. NMR samples were analyzed on Bruker Avance III HD 400 MHz or Avance III 500 MHz spectrometers. ^{1}H and ^{13}C NMR chemical shifts are given with reference to residual solvent resonances relative to $SiMe_4$. ^{31}P NMR chemical shifts are referenced externally to 85% aqueous H_3PO_4 (a sealed capillary tube containing 85% aqueous H_3PO_4 was inserted into each sample tube subject to ^{31}P NMR analysis). UV-Vis spectra were recorded at 24 °C using a CARY 100 Bio UV-Vis spectrophotometer.

Melting points are uncorrected and were determined for samples in capillary tubes sealed under argon. Elemental analyses of samples of **5** – **8** were carried out by Micro-Analysis, Inc., Wilmington, Delaware. High-resolution mass-spectral data were obtained in the Mass-spectrometry facility at the University of Kansas. 2-Formamido-6-bromo-1,3-diethoxycarbonylazulene,[1] 2-isocyano-1,3-diethoxycarbonylazulene,[2] 6-mercapto-1,3-diethoxycarbonylazulene,[3] and 6-mercapto-2-chloro-1,3-diethoxycarbonylazulene[3] were prepared according to our previously published procedures. Other reagents were obtained from commercial sources and used as received.

A2. Synthesis of compound 4. Ethyl-3-mercaptopropionate (0.139 mL, 1.13 mmol) was added to a solution of 2-formamido-6-bromo-1,3-diethoxycarbonylazulene (0.4036 g, 1.024 mmol) in 50 mL of pyridine. The reaction mixture was refluxed for 4 hours with stirring, then cooled to room temperature and concentrated to dryness under reduced pressure. The residue was subject to column chromatography on silica with neat CH_2Cl_2 as the eluent. The orange band was collected. Recrystallization of the product at -20 °C by layering pentane over its solution in a minimum amount of CH_2Cl_2 gave persimmon-coloured microcrystalline **4** (0.4224 g, 0.9439 mmol) in a 92% yield. Mp: 117-119 °C. HRMS (m/z, ES+): found for $[M+H]^+$ 448.1443; calcd for $C_{22}H_{26}NO_7S^+$ 448.1425. ^{1}H NMR ($CDCl_3$, 500 MHz, 25 °C): δ 1.27 (t, $^{3}J_{HH}$= 7.1 Hz, 3H, CH_3), 1.43 (t, $^{3}J_{HH}$ = 7.1 Hz, 6H, CH_3), 2.76 (t, $^{3}J_{HH}$ = 7.3 Hz, 2H, CH_2), 3.39 (t, $^{3}J_{HH}$ = 7.3 Hz, 2H, CH_2), 4.18 (q, $^{3}J_{HH}$ = 7.1 Hz, 2H, CH_2), 4.44 (q, $^{3}J_{HH}$ = 7.1 Hz, 4H, CH_2), 7.52 (d, $^{3}J_{HH}$ = 10.9 Hz, 2H, $H^{5,7}$), 8.61 (br s, 1H, N*H*), 9.11 (br d, $^{3}J_{HH}$ = 10.9 Hz, 2H, $H^{4,8}$), δ 10.12 (s, br, 1H, C*H*O) ppm. $^{13}C\{^{1}H\}$ NMR ($CDCl_3$, 126 MHz, 25 °C): δ 14.21 (CH_3), 14.42 (CH_3), 28.18 (SCH_2CH_2), 33.17 (SCH_2CH_2), 60.78 (CH_2CH_3), 61.17 (CH_2CH_3), 106.63 br, 128.19 br, 134.55, 139.54, 146.25 br, 152.84 (azulenic *C*), ~164.3 br

(NH*C*HO), 165.38 (*C*O_2Et), 171.07 (*C*O_2Et) ppm. UV-Vis (CH_2Cl_2, λ_{max} ($\varepsilon \times 10^{-3}$ M^{-1} cm^{-1}), 24 °C): 244 (16.3), 269 (17.3), ~275 (16.7) sh, 343 (46.1), 411 (20.9), 478 (1.01) nm.

A3. Synthesis of compound 5. Neat $POCl_3$ (57.3 μL, 0.642 mmol) was added to a cold (-95 °C) solution of **4** (0.2499 g, 0.5584 mmol) in 50 mL of CH_2Cl_2. After stirring for 10 min, the reaction mixture was treated with diisopropylamine (0.30 mL, 2.1 mmol), stirred for an additional 30 min at -95 °C, and then allowed to warm to room temperature over a period of 18 hours. After quenching the mixture with 100 mL of aqueous 10% Na_2CO_3, the organic layer was separated, washed with distilled water (3×50 mL), and concentrated to dryness under reduced pressure. The residue was recrystallized at -20 °C by layering pentane over a solution of the product in a minimum amount of CH_2Cl_2 to afford peach-red microcrystalline **5** (0.2271 g, 0.5281 mmol) in a 92% yield. Mp: 108-111 °C. Anal. calcd for $C_{22}H_{23}NO_6S$: C, 61.52; H, 5.40; N, 3.26. Found: C, 61.51; H, 5.50; N, 3.41. IR (CH_2Cl_2): $\nu_{N\equiv C}$ 2126 m, $\nu_{C=O}$ 1732 m, 1690 m cm^{-1}. ^1H NMR (500 MHz, CD_2Cl_2, 25 °C): δ 1.26 (t, $^3J_{HH}$ = 7.1 Hz, 3H, C*H*$_3$), 1.47 (t, $^3J_{HH}$ = 7.1 Hz, 6H, C*H*$_3$), 2.81 (t, $^3J_{HH}$ = 7.3 Hz, 2H, C*H*$_2$), 3.45 (t, $^3J_{HH}$ = 7.3 Hz, 2H, C*H*$_2$), 4.18 (q, $^3J_{HH}$ = 7.1 Hz, 2H, C*H*$_2$), 4.46 (q, $^3J_{HH}$ = 7.1 Hz, 4H, C*H*$_2$), 7.60 (d, $^3J_{HH}$ = 11.5 Hz, 2H, $H^{5,7}$), 9.43 (d,$^3J_{HH}$ = 11.5 Hz, 2H, $H^{4,8}$) ppm. $^{13}C\{^1H\}$ NMR ($CDCl_3$, 126 MHz, 25 °C): δ 14.34 (*C*H_3), 14.37 (*C*H_3), 28.10 (S*C*H_2CH_2), 32.99 (SCH_2*C*H_2), 61.12 (*C*H_2CH_3), 61.48 (*C*H_2CH_3), 113.41, 127.84, 138.31, 138.55, 159.69 (azulenic *C*), 163.61 (*C*O_2Et), 170.93 (*C*O_2Et), 176.29 (*C*NR) ppm. ^{14}N NMR (36 MHz, CD_2Cl_2, 25 °C): δ 173.50 ppm. UV-Vis (CH_2Cl_2, λ_{max} ($\varepsilon \times 10^{-3}$ M^{-1} cm^{-1}), 24 °C): 237 (23.1), 263 (17.4), 277 (13.7), 291 (13.7), 344 (42.8), ~369 (15.7) sh, 400 (28.4), 484 (1.55) nm.

A4. Synthesis of compound 6. A red-orange solution of $Cr(CO)_5$(THF) was prepared *in situ* by photolysis of $Cr(CO)_6$ (0.2052 g, 0.9320 mmol) dissolved in 150 mL of THF using a Hanovia Hg 450 W immersion lamp. Upon completion of the photolysis as judged by IR of the mixture in the $\nu_{C\equiv O}$ region, a solution of **5** (0.3851 g, 0.8967 mmol) in 50 mL of THF was added via cannula to the $Cr(CO)_5$(THF) solution at 0 °C. The resulting mixture was warmed to room temperature and stirred for 15 hours. The reactor was then opened to air and its content was concentrated to dryness under vacuum. The residue was passed through a short silica gel column using neat CH_2Cl_2. The first eluted band, vibrant orange in colour, provided an orange oily residue after solvent removal and drying at 10^{-2} torr. The oil was then triturated in pentane to afford an orange powder of **6** (0.3524 g, 0.5670 mmol) in a 63% yield. Mp: 62-65 °C. Anal. calcd for $C_{27}H_{23}CrNO_{11}S$: C, 52.18; H, 3.73; N, 2.25. Found: C, 52.15; H, 3.82; N, 2.36. IR (CH_2Cl_2): $\nu_{N\equiv C}$ 2140 m, $\nu_{C\equiv O}$ 2050 s ($A_1^{(1)}$), 2000 vw (B_1), 1958 vs ($A_1^{(2)}$ + E), $\nu_{C=O}$ 1732 m, 1691 m cm^{-1}. ^1H NMR (500 MHz, $CDCl_3$, 25 °C): δ 1.29 (t, $^3J_{HH}$ = 7.1 Hz, 3H, C*H*$_3$), 1.48 (t, $^3J_{HH}$ = 7.1 Hz, 6H, C*H*$_3$), 2.81 (t, $^3J_{HH}$ = 7.5 Hz, 2H, C*H*$_2$), 3.44 (t, $^3J_{HH}$ = 7.5 Hz, 2H, CH_2), 4.21 (q, $^3J_{HH}$ = 7.1 Hz, 2H, C*H*$_2$), 4.55 (q, $^3J_{HH}$ = 7.1 Hz, 4H, C*H*$_2$), 7.59 (d, $^3J_{HH}$ = 11.5 Hz, 2H, $H^{5,7}$), 9.52 (d,$^3J_{HH}$ = 11.5 Hz, 2H, $H^{4,8}$) ppm. $^{13}C\{^1H\}$ NMR ($CDCl_3$, 126 MHz, 25 °C): δ 14.33 (*C*H_3), 14.84 (*C*H_3), δ 28.09 (S*C*H_2CH_2), 33.02 (SCH_2*C*H_2), 60.49 (*C*H_2CH_3), 61.46 (*C*H_2CH_3), 113.20, 129.80, 138.05, 138.83, 158.96 (azulenic *C*), 163.53 (*C*O_2Et), 170.94 (*C*O_2Et), 181.72 (*C*NR), 214.68 (Cr*C*O, *cis*), 216.85 (Cr*C*O, *trans*) ppm. UV-Vis (CH_2Cl_2, λ_{max} ($\varepsilon \times 10^{-3}$ M^{-1} cm^{-1}), 24 °C): 235 (60.3), 278 (29.9), 344 (44.7), 454 (31.2) nm.

A5. Synthesis of compound 7. Absolute ethanol, deionized H_2O, and 3M aqueous H_2SO_4 were all purged with argon for 1 hour prior to their use in the following procedure. A solution of sodium ethoxide prepared by *carefully* dissolving sodium metal (0.0466 g, 2.027 mmol) in 10 mL of ethanol was transferred into a flask containing a suspension of **6** (0.6304 g, 1.014 mmol) in 100 mL of ethanol. The reaction mixture gradually acquired red colour while being stirred for 4 hours at room temperature. Then, it was diluted with 300 mL of H_2O and slowly acidified with 3M aqueous H_2SO_4 until precipitation of a deep red solid. This precipitate was filtered off, washed extensively with water and dried at 10^{-2} torr. The product was recrystallized at -20 °C by layering pentane over its solution in a minimum amount of CH_2Cl_2 to afford small auburn red crystals of **7** (0.4248 g, 0.8147 mmol) in an 80% yield. Mp: 157 °C (dec). Anal. calcd for $C_{22}H_{15}CrNO_9S$: C, 50.68; H, 2.90; N 2.69. Found: C, 50.57, H, 2.98; N, 2.75. IR (CH_2Cl_2): $\nu_{S\text{-}H}$ 2583 vw, $\nu_{N\equiv C}$ 2140 m, $\nu_{C\equiv O}$ 2049 s ($A_1^{(1)}$), 2000 vw (B_1), 1958 vs ($A_1^{(2)}$ + E), $\nu_{C=O}$ 1692 m cm^{-1}. IR (KBr): $\nu_{S\text{-}H}$ 2534 vw, $\nu_{N\equiv C}$ 2137 m, $\nu_{C\equiv O}$ 2051 s, 2008 vw, 1977 s sh, 1954 vs, 1937 vs, $\nu_{C=O}$ 1683 m, 1673 m cm^{-1}. ^{1}H NMR (500 MHz, $CDCl_3$, 25 °C): δ 1.51 (t, $^3J_{HH}$ = 7.1 Hz, 6H, CH_3), 4.35 (s, 1H, S*H*), 4.58 (q, $^3J_{HH}$ = 7.1 Hz, 4H, CH_2), 7.68 (d, $^3J_{HH}$ = 11.2 Hz, 2H, $H^{5,7}$), 9.51 (d, $^3J_{HH}$= 11.2 Hz, 2H, $H^{4,8}$) ppm. $^{13}C\{^1H\}$ NMR ($CDCl_3$, 126 MHz, 25 °C): δ 14.84 (CH_3), 61.07 (CH_2), 113.43, 130.09, 130.54, 138.48, 139.31, 155.09 (azulenic *C*), 163.48 (*C*O_2Et), 182.27 (*C*NR), 214.65 (Cr*C*O, *cis*), 216.77 (Cr*C*O, *trans*) ppm. ^{14}N NMR (36 MHz, CD_2Cl_2, 25 °C): δ 180.84 ppm. UV-Vis (CH_2Cl_2, λ_{max} ($\varepsilon\times10^{-3}$ M^{-1} cm^{-1}), 24 °C): 233 (62.8), 277 (28.1), 333 (57.2), ~360 (24.8) sh, 452 (25.8) nm.

A6. Synthesis of compound 8. All manipulations in the following procedure were conducted with protection from ambient laboratory lighting. A solid mixture of Me_2SAuCl (0.0566 g, 0.1922 mmol) and PPh_3 (0.0504 g, 0.1922 mmol) was dissolved in 20 mL of CH_2Cl_2 and the resulting solution was stirred for 1 hour at room temperature. The solvent was removed under vacuum and the residue was washed with 5 mL of pentane before being combined with compound **7** (0.1012 g, 0.1941 mmol) and solid NaOH (0.0462 g, 1.152 mmol) under a flow of argon. The resulting mixture was dissolved in 10 mL of methanol, stirred for 10 minutes, diluted with 10 mL of CH_2Cl_2, and continued to be stirred for 15 hours. Then, all solvent was removed under vacuum and the residue was re-dissolved in 50 mL of CH_2Cl_2 and washed with deionized water (3×25 mL). The CH_2Cl_2 layer was separated, concentrated under reduced pressure, and passed through a 45cm-tall (2 cm ID) silica gel column using neat CH_2Cl_2 as eluent. The first bright orange band was collected. The product was recrystallized by carefully layering pentane over its solution in CH_2Cl_2 to afford orange-red crystals of **8** (0.1638 g, 0.1672 mmol), which were dried at 10^{-2} torr, in an 86% yield. Mp: 140-143 °C. Anal. calcd for $C_{40}H_{29}CrNO_9S$: C, 49.04; H 2.98; N 1.43. Found: C, 49.10; H, 3.08; N, 1.48. IR (CH_2Cl_2): $\nu_{N\equiv C}$ 2144 m, $\nu_{C\equiv O}$ 2054 s ($A_1^{(1)}$), 2003 vw (B_1), 1957 vs ($A_1^{(2)}$ + E), $\nu_{C=O}$ 1685 m cm^{-1}. IR (KBr): $\nu_{N\equiv C}$ 2145 m, $\nu_{C\equiv O}$ 2054 s, 1996 vw, 1951 vs br, 1935 vs sh, 1924 s sh, $\nu_{C=O}$ 1692 m cm^{-1}. ^{1}H NMR (500 MHz, $CDCl_3$, 25 °C): δ 1.46 (t, $^3J_{HH}$ = 7.1 Hz, 6H, CH_3), 4.51 (q, $^3J_{HH}$= 7.1 Hz, 4H, CH_2), 7.50-7.63 (m, 15H, PPh_3), 8.29 (d, $^3J_{HH}$ = 11.5 Hz, 2H, $H^{5,7}$), 9.18 (d,$^3J_{HH}$ = 11.5 Hz, 2H, $H^{4,8}$) ppm. $^{13}C\ \{^1H\}$ NMR ($CDCl_3$, 126 MHz, 25 °C): δ 14.90 (CH_3), 60.58 (CH_2), 112.17, 127.31 (azulenic *C*), 128.59 (d, $^1J_{CP}$ = 58.5 Hz, *i-C*, Ph), 129.54 (d, $^3J_{CP}$ = 11.6 Hz, *o-C*, Ph), 132.32 (d, $^4J_{CP}$ = 2.6 Hz, *p-*

C, Ph), 134.14 (d, $^2J_{CP}$= 13.7 Hz, *m-C*, Ph), 136.11, 136.28, 138.38 (azulenic *C*), 163.98 (*C*O_2Et), 174.18 (azulenic C^2), 178.55 (*C*NR), 214.91 (Cr*C*O, *cis*), 217.26 (Cr*C*O, *trans*) ppm. $^{31}P\{^1H\}$ NMR (162 MHz, CD_2Cl_2, 22 °C): 38.53 ppm. UV-Vis (CH_2Cl_2, λ_{max} ($\varepsilon\times10^{-3}$ M^{-1} cm^{-1}), 24 °C): 232 (75.5), 276 (32.6), 344 (29.2), 359 (28.7), 469 (53.8) nm.

A7. Synthesis of compound 11. Red microcrystalline **11** was prepared from $Cr(CO)_6$ and 2-isocyano-1,3-diethoxycarbonylazulene following the protocol established for the preparation of **6** from **5**. IR (CH_2Cl_2): $\nu_{N\equiv C}$ 2140 m, $\nu_{C\equiv O}$ 2049 s, 1959 vs, $\nu_{C=O}$ 1691 m cm^{-1}. IR (KBr): $\nu_{N\equiv C}$ 2140 m, $\nu_{C\equiv O}$ 2053 s, 1976 s sh, 1960 vs, 1936 vs, $\nu_{C=O}$ 1686 m cm^{-1}. ^1H NMR (400 MHz, $CDCl_3$, 25 °C): δ 1.50 (t, $^3J_{HH}$ = 7.2 Hz, 6H, CH_3), 4.58 (q, $^3J_{HH}$ = 7.1 Hz, 4H, CH_2), 7.85 (t, $^3J_{HH}$ = 10.0 Hz, 2H, $H^{5,7}$), 9.68 (t, $^3J_{HH}$ = 9.7 Hz, 1H, H^6), 9.85 (d, $^3J_{HH}$ = 10.3 Hz, 2H, $H^{4,8}$) ppm. $^{13}C\{^1H\}$ NMR ($CDCl_3$, 126 MHz, 25 °C): δ 14.86 (*C*H_3), 61.09 (*C*H_2CH_3), 112.48, 131.82, 132.37, 140.70, 142.01, 142.15 (azulenic *C*), 163.52 (*C*O_2Et), 183.36 (*C*NR), 214.60 (Cr*C*O, *cis*), 216.69 (Cr*C*O, *trans*) ppm. UV-Vis (CH_2Cl_2, λ_{max} ($\varepsilon\times10^{-3}$ M^{-1} cm^{-1}), 24 °C): 237 (115.3), 299 (105), 356 (35.9), 441 (32.8) nm.

A8. Synthesis of compound 12. Bright pink microcrystalline 2-isocyano-6-bromo-1,3-diethoxycarbonylazulene was synthesized by dehydrating 2-formamido-6-bromo-1,3-diethoxycarbonylazulene with $POCl_3$ following the procedure established for the synthesis of **5**. Bright orange microcrystalline **12** was prepared from $Cr(CO)_6$ and 2-isocyano-1,3-diethoxycarbonylazulene following the protocol established for the preparation of **6** from **5**.

2-Isocyano-6-bromo-1,3-diethoxycarbonylazulene: Mp: 174-176 °C (dec). ^1H NMR ($CDCl_3$, 500 MHz, 25 °C): δ 1.51 (t, $^3J_{HH}$= 7.1 Hz, 6 H, CH_3), 4.51 (q, $^3J_{HH}$= 7.1 Hz, 4 H, CH_2), 8.13 (d, $^3J_{HH}$= 11. Hz, 2 H, C$H^{5,7}$), 9.54 (d, $^3J_{HH}$= 11. Hz, 2 H, C$H^{4,8}$) ppm. $^{13}C\{^1H\}$ NMR ($CDCl_3$, 126 MHz, 25 °C): δ 14.33 (*C*H_3), 61.42 (*C*H_2), 100.12, 114.07, 135.50, 139.16, 139.95, 141.22 (azulenic *C*), 163.22 (*C*O_2Et) 178.01 (*C*N) ppm. IR (CH_2Cl_2): $\nu_{N\equiv C}$ 2126, $\nu_{C=O}$ 1695 m cm^{-1}. UV-Vis (CH_2Cl_2, λ_{max} ($\varepsilon\times10^{-3}$ M^{-1} cm^{-1}), 24 °C): 274 (44.9), 283 (43.7), 362 (12.7), 523 (0.9).

12: Mp: 192-194 °C (dec). ^1H NMR ($CDCl_3$, 500 MHz, 25 °C): δ 1.49 (t, $^3J_{HH}$= 7.1 Hz, 6 H, CH_3), 4.57 (q, $^3J_{HH}$= 7.1 Hz, 4 H, CH_2), 8.11 (d, $^3J_{HH}$= 12. Hz, 2 H, C$H^{5,7}$), 9.53 (d, $^3J_{HH}$= 12. Hz, 2 H, C$H^{4,8}$) ppm. $^{13}C\{^1H\}$ NMR ($CDCl_3$, 126 MHz, 25 °C): δ 14.81 (*C*H_3), 61.30 (*C*H_2), 113.85, 131.87, 135.70, 138.45, 140.35, 140.51 (azulenic *C*), 163.21 (*C*O_2Et) 184.42 (*C*N), 214.47 (*C*O_{eq}), 216.52 (*C*O_{ax}) ppm. IR (CH_2Cl_2): $\nu_{N\equiv C}$ 2138 m, $\nu_{C\equiv O}$ 2047 s, 1960 vs, $\nu_{C=O}$ 1695 m cm^{-1}. UV-Vis (CH_2Cl_2, λ_{max} ($\varepsilon\times10^{-3}$ M^{-1} cm^{-1}), 24 °C): 240 (48.1), 282 (24.0), 314 (54.4), 341 (17.1), 364 (14.0), 459 (16.6).

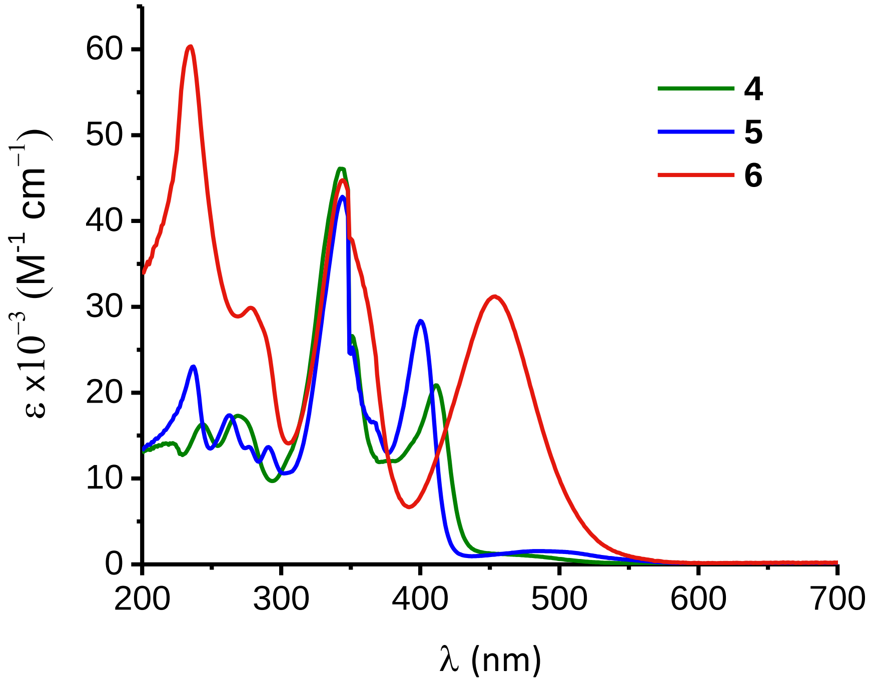

Figure S1. UV-Vis spectra of **4**, **5**, and **6** in CH_2Cl_2.

B. X-RAY STRUCTURE DETERMINATION FOR 8·$^{3}/_{4}CH_2Cl_2$

B1. Experimental

Red-orange, X-ray quality crystals of **8**·$^{3}/_{4}CH_2Cl_2$ were grown by carefully layering pentane over a solution of this compound in CH_2Cl_2 at room temperature and then cooling the sample to -20 °C for a period of one week. A suitable single crystal of **8** was selected using a MiTeGen thin plastic crystal holder and mounted on the copper-pin attached to the goniometer head of a Bruker APEX 2 diffractometer equipped with a SMART CCD area detector. All intensity data were collected in the ω scan mode using a Mo tube (Kα radiation; λ = 0.71073 Å) with a highly oriented graphite monochromator. A total of 1456 frames were obtained with the overall crystal exposure time of 9.07 hours. The frames were integrated using the narrow-frame algorithm of the Bruker SAINT software package.[4] The crystal faces were indexed with the aid of a videomicroscope (Figure S1). The data were corrected empirically (SADABS) for absorption effects.[4] The structure was solved by direct methods and refined by least squares on weighted F^2 values for all reflections using the Bruker SHELXTL Software Package.[5] The key crystal data and refinement information is provided in Table S1. The largest difference peak and hole are located near Au1 (1.17 Å; x = 0.8136, y = 0.3497, z = 0.7120) and Au2 (0.82 Å; x = 0.7614, y = 0.3244, z = 0.8185), respectively. Both likely represent "ripples" of electron density typical for structures containing multiple heavy atoms.

With the exception of a disordered CH_2Cl_2 solvent molecule of crystallization (*vide infra*), all H-atoms in the structure were geometrically attached to the corresponding host carbon atoms (using their idealized hybridization status) and refined isotropically. Non-hydrogen atoms were refined with anisotropic displacement parameters. All thermal ellipsoids are drawn at the 50% probability level. Molecular structure drawings were obtained using the ORTEP 3v2[6] freeware package.

The asymmetric unit of the crystal structure of **8**·$^{3}/_{4}CH_2Cl_2$ features two independent molecules of **8** linked together via a weak aurophilic interaction (Figures S2 and S3). Two ethoxycarbonyl substituents exhibit positional disorders which were successfully modeled by splitting the OEt unit attached to C14 into two individual contributors O4-C15-C16 and O4A-C15A-C16A (Figure S4, left) as well as by resolving the methyl group of the OEt unit attached to C49 into two alternative positions C51 and C51a (Figure S4, right). In addition to the pair of the heterobimetallic dimers, the entire unit cell of **8**·$^{3}/_{4}CH_2Cl_2$ contains three disordered CH_2Cl_2 molecules of crystallization. One of these CH_2Cl_2 molecules is disordered into two positions of its CH_2 moiety via the inversion center as shown in Figure S5, left. The other two symmetry related CH_2Cl_2 solvent molecules were resolved into two positions via rotation around the C1S-Cl1S bond as illustrated in Figure S5, right. The H-atoms of the latter disordered CH_2Cl_2 could not be attached to the carbon atom C1S in a meaningful way so these two hydrogen atoms were left out from the refinement. Crystal data, data collection, solution, and refinement information for **8**·$^{3}/_{4}CH_2Cl_2$ are provided in Table S1. Tables S2 through S5 contain atomic coordinates as well as displacement and metric parameters for **8**. This crystal structure was deposited into the CCDC under the number *1410785*.

Table S1. Crystal data and structure refinement for **8**·$^{3}/_{4}CH_2Cl_2$.

Chemical formula	$C_{40.75}H_{30.5}AuCl_{1.50}NO_9PS$	
Formula weight	1043 g/mol	
Temperature	120(2) K	
Wavelength	0.71073 Å	
Crystal system	Triclinic	
Space group	$P\bar{1}$	
Unit cell dimensions	*a* = 13.4225(14) Å	α = 74.619(2)°
	b = 14.2249(15) Å	β = 89.572(2)°
	c = 24.728(3) Å	γ = 62.1260(10)°
Volume	3986.3(7) $Å^3$	
Z	4	
Density (calculated)	1.738 g cm^{-3}	
Absorption coefficient	4.198 mm^{-1}	
F(000)	2054	
Crystal size	0.098 × 0.132 × 0.299 mm^3	
Crystal habit	clear light red-orange block	
Theta range for data collection	1.66° to 30.56°	
Index ranges	$-19 \le h \le 19$, $-20 \le k \le 20$, $-35 \le l \le 35$	
Reflections collected	61888	
Independent reflections	24204 [R_{int} = 0.0575]	
Coverage of independent reflections	99.0%	
Absorption correction	multi-scan	
Max. and min. transmission	0.7455 and 0.5006	
Refinement method	Full-matrix least-squares on F^2	
Data / restraints / parameters	24204 / 119 / 1072	
Goodness-of-fit on F^2	1.041	
Δ/σ_{max}	0.003	
Final R indices [15125 data, I>2σ(I)]	R_1 = 0.0469, wR_2 = 0.0843	
R indices (all data)	R_1 = 0.0954, wR_2 = 0.1054	
Weighting scheme	$w = 1/[\sigma^2(F_o^2)+(0.0328P)^2+5.2506P]$, where $P = (F_o^2+2F_c^2)/3$	
Largest diff. peak and hole	1.792 and -2.344 $eÅ^{-3}$	
R.M.S. deviation from mean	0.197 $eÅ^{-3}$	

$R_1 = \Sigma \| F_o| - |F_c\| / \Sigma |F_o|$

$wR_2 = \{ \Sigma [w(F_o^2 - F_c^2)^2] / \Sigma [w(F_o^2)^2] \}^{1/2}$

Table S2. Atomic coordinates and equivalent isotropic displacement parameters for **8**·$^{3}/_{4}CH_2Cl_2$. U(eq) is defined as one third of the trace of the orthogonalized U_{ij} tensor.

	**x/a**	**y/b**	**z/c**	**U(eq)**
Au1	0.80382(2)	0.32181(2)	0.67269(2)	0.02468(6)
Au2	0.77970(2)	0.36323(2)	0.79470(2)	0.02599(6)
C1	0.8101(5)	0.0863(4)	0.7436(2)	0.0242(11)
C2	0.9263(4)	0.0487(4)	0.7406(2)	0.0256(11)
C3	0.0176(5)	0.9446(4)	0.7634(2)	0.0256(11)
C4	0.0230(5)	0.8440(4)	0.7946(2)	0.0254(11)
C5	0.9272(5)	0.8234(4)	0.8055(2)	0.0246(11)
C6	0.8121(5)	0.9036(5)	0.7903(2)	0.0322(13)
C7	0.7611(5)	0.0169(4)	0.7651(2)	0.0292(12)
C8	0.1238(5)	0.7428(4)	0.8189(2)	0.0276(12)
C9	0.2392(5)	0.7280(4)	0.8211(2)	0.0335(13)
C10	0.5090(6)	0.4885(5)	0.8920(3)	0.063(2)
C11	0.4339(5)	0.6056(5)	0.8571(3)	0.0451(16)
C12	0.0899(5)	0.6621(4)	0.8425(2)	0.0285(12)
C13	0.9717(5)	0.7091(4)	0.8352(2)	0.0300(13)
C14	0.9126(6)	0.6441(5)	0.8561(3)	0.0367(14)
C17	0.2165(5)	0.4545(5)	0.8875(2)	0.0325(13)
C18	0.1999(6)	0.3038(5)	0.9717(3)	0.0422(16)
C19	0.1873(5)	0.2987(4)	0.8648(2)	0.0336(13)
C20	0.3738(5)	0.1341(5)	0.9378(2)	0.0357(14)
C21	0.4106(6)	0.2837(5)	0.9654(3)	0.0486(17)
C22	0.3881(6)	0.2936(5)	0.8561(3)	0.0416(15)
C23	0.8704(4)	0.3682(4)	0.5428(2)	0.0228(11)
C24	0.8450(4)	0.4458(4)	0.4900(2)	0.0274(12)
C25	0.8278(5)	0.4219(4)	0.4414(2)	0.0300(12)
C26	0.8377(5)	0.3192(5)	0.4448(2)	0.0311(13)
C27	0.8660(5)	0.2390(4)	0.4971(2)	0.0324(13)
C28	0.8819(5)	0.2637(4)	0.5461(2)	0.0289(12)
C29	0.8261(4)	0.5480(4)	0.5869(2)	0.0220(10)
C30	0.8941(5)	0.5992(4)	0.5779(2)	0.0246(11)
C31	0.8450(5)	0.7141(4)	0.5569(2)	0.0311(13)
C32	0.7282(5)	0.7787(4)	0.5444(2)	0.0327(13)
C33	0.6596(5)	0.7287(4)	0.5537(2)	0.0332(13)
C34	0.7078(5)	0.6145(4)	0.5749(2)	0.0286(12)
C35	0.0390(4)	0.3437(4)	0.6263(2)	0.0243(11)

	x/a	y/b	z/c	U(eq)
C36	0.1147(5)	0.3134(4)	0.5879(2)	0.0291(12)
C37	0.2289(5)	0.2771(4)	0.6021(3)	0.0360(14)
C38	0.2673(5)	0.2721(5)	0.6546(3)	0.0385(14)
C39	0.1928(5)	0.3017(5)	0.6938(3)	0.0386(15)
C40	0.0792(5)	0.3353(4)	0.6798(2)	0.0323(13)
C41	0.7684(4)	0.6000(4)	0.7223(2)	0.0235(11)
C42	0.7148(4)	0.7140(4)	0.6915(2)	0.0247(11)
C43	0.7590(4)	0.7868(4)	0.6742(2)	0.0233(11)
C44	0.8708(4)	0.7669(4)	0.67971(19)	0.0197(10)
C45	0.9696(4)	0.6607(4)	0.7081(2)	0.0190(10)
C46	0.9703(4)	0.5623(4)	0.7392(2)	0.0229(11)
C47	0.8834(4)	0.5361(4)	0.7455(2)	0.0248(11)
C48	0.9115(4)	0.8442(4)	0.6572(2)	0.0200(10)
C49	0.8504(4)	0.9633(4)	0.6259(2)	0.0236(11)
C50	0.6749(5)	0.1269(4)	0.5886(3)	0.0384(14)
C51	0.5500(13)	0.1701(14)	0.5971(14)	0.040(5)
C51A	0.5585(13)	0.1547(14)	0.5711(12)	0.043(5)
C52	0.0299(4)	0.7853(4)	0.6703(2)	0.0193(10)
C53	0.0685(4)	0.6735(4)	0.7012(2)	0.0210(10)
C54	0.1862(4)	0.5857(4)	0.7223(2)	0.0249(11)
C55	0.3812(5)	0.5396(5)	0.7232(3)	0.0461(17)
C56	0.4215(6)	0.4705(6)	0.6847(3)	0.068(2)
C57	0.1675(4)	0.8593(4)	0.6407(2)	0.0248(11)
C58	0.1812(5)	0.0001(5)	0.5444(3)	0.0331(13)
C59	0.1960(5)	0.0240(4)	0.6514(2)	0.0315(13)
C60	0.3803(5)	0.9634(5)	0.5895(2)	0.0327(13)
C61	0.3768(5)	0.8256(5)	0.6846(3)	0.0330(13)
C62	0.3555(5)	0.7884(5)	0.5866(3)	0.0329(13)
C63	0.8457(5)	0.2638(4)	0.9344(2)	0.0290(12)
C64	0.7915(6)	0.2323(5)	0.9775(3)	0.0438(16)
C65	0.7696(7)	0.2773(6)	0.0219(3)	0.058(2)
C66	0.8011(6)	0.3547(5)	0.0236(3)	0.0492(18)
C67	0.8589(6)	0.3858(5)	0.9816(3)	0.0435(16)
C68	0.8790(5)	0.3426(5)	0.9367(3)	0.0392(14)
C69	0.8227(5)	0.1088(4)	0.8872(2)	0.0241(11)
C70	0.9002(5)	0.9955(4)	0.9083(2)	0.0299(12)
C71	0.8610(5)	0.9185(4)	0.9232(2)	0.0305(12)
C72	0.7465(5)	0.9537(4)	0.9170(2)	0.0315(13)
C73	0.6693(5)	0.0660(5)	0.8952(2)	0.0338(13)

	x/a	y/b	z/c	U(eq)
C74	0.7074(5)	0.1427(4)	0.8801(2)	0.0310(13)
C75	0.0215(5)	0.1344(4)	0.8767(2)	0.0292(12)
C76	0.0918(5)	0.0846(5)	0.9281(3)	0.0357(14)
C77	0.2065(6)	0.0136(5)	0.9308(3)	0.0471(17)
C78	0.2507(6)	0.9902(5)	0.8829(3)	0.0505(18)
C79	0.1816(6)	0.0403(6)	0.8318(3)	0.0534(19)
C80	0.0676(5)	0.1139(5)	0.8281(3)	0.0387(14)
C2S	0.4413(10)	0.5060(11)	0.5085(5)	0.044(3)
C1S	0.4189(6)	0.0025(7)	0.7597(3)	0.063(2)
Cl1S	0.51558(19)	0.97889(18)	0.71122(9)	0.0713(6)
Cl2S	0.4450(5)	0.0688(6)	0.8048(2)	0.0781(18)
Cl2A	0.4997(16)	0.995(2)	0.8208(5)	0.098(5)
Cl3S	0.42855(18)	0.57038(15)	0.44552(10)	0.0783(7)
Cr1	0.29847(8)	0.29147(7)	0.91529(4)	0.0331(2)
Cr2	0.27728(7)	0.91154(7)	0.61546(4)	0.02656(19)
N1	0.1630(4)	0.5501(4)	0.86885(19)	0.0338(11)
N2	0.1003(3)	0.8313(3)	0.65400(17)	0.0231(9)
O1	0.2640(3)	0.8010(3)	0.80187(19)	0.0455(11)
O2	0.3187(3)	0.6233(3)	0.84944(17)	0.0372(10)
O3	0.9584(4)	0.5474(3)	0.88427(18)	0.0469(11)
O5	0.4423(4)	0.2947(4)	0.8201(2)	0.0564(13)
O6	0.4216(4)	0.0391(3)	0.95049(18)	0.0496(12)
O7	0.1194(4)	0.3076(3)	0.83338(18)	0.0461(11)
O8	0.1389(4)	0.3159(4)	0.0055(2)	0.0589(13)
O9	0.4789(5)	0.2771(5)	0.9960(2)	0.0776(18)
O10	0.8967(3)	0.0191(3)	0.60977(17)	0.0375(10)
O11	0.7374(3)	0.0070(3)	0.61755(16)	0.0302(9)
O12	0.2152(3)	0.4914(3)	0.75179(16)	0.0329(9)
O13	0.2612(3)	0.6216(3)	0.70608(17)	0.0348(9)
O14	0.4041(4)	0.7135(4)	0.5700(2)	0.0496(12)
O15	0.4389(3)	0.7762(3)	0.72560(18)	0.0434(11)
O16	0.4443(4)	0.9918(4)	0.57311(18)	0.0458(11)
O17	0.1484(4)	0.0841(3)	0.67643(18)	0.0430(11)
O18	0.1263(4)	0.0522(4)	0.50175(19)	0.0457(11)
P1	0.88680(11)	0.39792(10)	0.60817(6)	0.0223(3)
P2	0.86925(12)	0.21338(11)	0.87274(6)	0.0259(3)
S1	0.71220(12)	0.22808(11)	0.72078(6)	0.0295(3)
S2	0.68049(11)	0.53858(11)	0.72935(6)	0.0283(3)
O4	0.8078(14)	0.7004(14)	0.8490(5)	0.033(3)

	**x/a**	**y/b**	**z/c**	**U(eq)**
C15	0.7398(11)	0.6434(11)	0.8706(6)	0.042(4)
C16	0.6190(10)	0.7330(10)	0.8534(7)	0.060(5)
O4A	0.7890(18)	0.7108(19)	0.8283(7)	0.030(3)
C15A	0.7211(16)	0.6592(17)	0.8467(8)	0.042(5)
C16A	0.6642(19)	0.6870(19)	0.8971(8)	0.060(6)

Table S3. Bond lengths [Å] for **8**·$^{3}/_{4}CH_2Cl_2$.

Au1-P1	2.2677(13)	Au1-S1	2.3184(13)
Au1-Au2	3.2102(4)	Au2-P2	2.2736(13)
Au2-S2	2.3226(13)	C1-C2	1.400(7)
C1-C7	1.420(7)	C1-S1	1.744(5)
C2-C3	1.372(7)	C2-H56	0.95
C3-C4	1.402(6)	C3-H57	0.95
C4-C8	1.413(7)	C4-C5	1.455(7)
C5-C6	1.401(8)	C5-C13	1.416(7)
C6-C7	1.377(7)	C6-H60	0.95
C7-H61	0.95	C8-C12	1.413(7)
C8-C9	1.462(8)	C9-O1	1.215(6)
C9-O2	1.349(6)	C10-C11	1.489(8)
C10-H69A	0.98	C10-H69B	0.98
C10-H69C	0.98	C11-O2	1.450(7)
C11-H68A	0.99	C11-H68B	0.99
C12-N1	1.387(7)	C12-C13	1.395(8)
C13-C14	1.475(7)	C14-O3	1.210(7)
C14-O4	1.235(18)	C14-O4A	1.52(2)
C17-N1	1.155(7)	C17-Cr1	1.960(6)
C18-O8	1.155(8)	C18-Cr1	1.901(8)
C19-O7	1.141(6)	C19-Cr1	1.900(6)
C20-O6	1.142(6)	C20-Cr1	1.891(6)
C21-O9	1.146(7)	C21-Cr1	1.901(7)
C22-O5	1.147(7)	C22-Cr1	1.891(7)
C23-C24	1.389(7)	C23-C28	1.401(6)
C23-P1	1.815(5)	C24-C25	1.382(7)
C24-H15	0.95	C25-C26	1.382(7)
C25-H14	0.95	C26-C27	1.393(7)
C26-H13	0.95	C27-C28	1.391(7)
C27-H27	0.95	C28-H28	0.95
C29-C30	1.395(7)	C29-C34	1.400(7)
C29-P1	1.812(5)	C30-C31	1.387(7)
C30-H30	0.95	C31-C32	1.380(8)
C31-H8	0.95	C32-C33	1.388(8)
C32-H9	0.95	C33-C34	1.380(7)
C33-H10	0.95	C34-H11	0.95
C35-C36	1.382(7)	C35-C40	1.388(7)

C35-P1	1.823(5)	C36-C37	1.383(8)
C36-H6	0.95	C37-C38	1.373(8)
C37-H5	0.95	C38-C39	1.388(8)
C38-H4	0.95	C39-C40	1.382(8)
C39-H3	0.95	C40-H2	0.95
C41-C47	1.401(7)	C41-C42	1.411(6)
C41-S2	1.752(5)	C42-C43	1.393(6)
C42-H16	0.95	C43-C44	1.393(7)
C43-H17	0.95	C44-C48	1.431(6)
C44-C45	1.453(6)	C45-C46	1.403(6)
C45-C53	1.426(6)	C46-C47	1.376(6)
C46-H35	0.95	C47-H36	0.95
C48-C52	1.398(7)	C48-C49	1.469(6)
C49-O10	1.211(6)	C49-O11	1.335(6)
C50-C51A	1.458(14)	C50-O11	1.465(6)
C50-C51	1.527(18)	C50-H20A	0.99
C50-H20B	0.99	C51-H51A	0.98
C51-H51B	0.98	C51-H51C	0.98
C51A-H51D	0.98	C51A-H51E	0.98
C51A-H51F	0.98	C52-N2	1.384(6)
C52-C53	1.405(6)	C53-C54	1.466(7)
C54-O12	1.216(6)	C54-O13	1.343(6)
C55-O13	1.458(6)	C55-C56	1.468(9)
C55-H32A	0.99	C55-H32B	0.99
C56-H33A	0.98	C56-H33B	0.98
C56-H33C	0.98	C57-N2	1.158(6)
C57-Cr2	1.969(5)	C58-O18	1.135(7)
C58-Cr2	1.918(7)	C59-O17	1.135(6)
C59-Cr2	1.910(6)	C60-O16	1.139(6)
C60-Cr2	1.887(5)	C61-O15	1.141(6)
C61-Cr2	1.898(6)	C62-O14	1.143(6)
C62-Cr2	1.900(6)	C63-C64	1.380(8)
C63-C68	1.400(7)	C63-P2	1.817(6)
C64-C65	1.376(8)	C64-H42	0.95
C65-C66	1.361(8)	C65-H41	0.95
C66-C67	1.393(9)	C66-H40	0.95
C67-C68	1.373(8)	C67-H39	0.95
C68-H38	0.95	C69-C74	1.386(7)
C69-C70	1.395(7)	C69-P2	1.820(5)
C70-C71	1.391(7)	C70-H44	0.95

C71-C72	1.371(7)	C71-H45	0.95
C72-C73	1.386(7)	C72-H46	0.95
C73-C74	1.377(7)	C73-H47	0.95
C74-H48	0.95	C75-C80	1.384(8)
C75-C76	1.388(7)	C75-P2	1.801(6)
C76-C77	1.381(8)	C76-H54	0.95
C77-C78	1.371(9)	C77-H53	0.95
C78-C79	1.374(9)	C78-H52	0.95
C79-C80	1.378(9)	C79-H51	0.95
C80-H50	0.95	C2S-Cl3S	1.547(12)
C2S-Cl3S	1.768(12)	C2S-H2S1	0.99
C2S-H2S2	0.99	C1S-Cl1S	1.737(7)
C1S-Cl2S	1.768(7)	C1S-Cl2A	1.819(12)
Cl3S-C2S	1.768(12)	O4-C15	1.493(17)
C15-C16	1.492(14)	C15-H15A	0.99
C15-H15B	0.99	C16-H16A	0.98
C16-H16B	0.98	C16-H16C	0.98
O4A-C15A	1.42(2)	C15A-C16A	1.502(16)
C15A-H15C	0.99	C15A-H15D	0.99
C16A-H16D	0.98	C16A-H16E	0.98
C16A-H16F	0.98		

Table S4. Bond angles [°] for **8**·$^{3}/_{4}CH_2Cl_2$.

P1-Au1-S1	165.82(5)	P1-Au1-Au2	120.98(4)
S1-Au1-Au2	73.19(4)	P2-Au2-S2	167.25(5)
P2-Au2-Au1	118.21(4)	S2-Au2-Au1	74.43(4)
C2-C1-C7	125.0(5)	C2-C1-S1	120.9(4)
C7-C1-S1	114.1(4)	C3-C2-C1	129.8(5)
C3-C2-H56	115.1	C1-C2-H56	115.1
C2-C3-C4	131.1(5)	C2-C3-H57	114.5
C4-C3-H57	114.5	C3-C4-C8	125.6(5)
C3-C4-C5	126.5(5)	C8-C4-C5	107.8(4)
C6-C5-C13	126.7(5)	C6-C5-C4	125.7(5)
C13-C5-C4	107.6(5)	C7-C6-C5	130.8(5)
C7-C6-H60	114.6	C5-C6-H60	114.6
C6-C7-C1	130.2(5)	C6-C7-H61	114.9
C1-C7-H61	114.9	C12-C8-C4	106.5(5)
C12-C8-C9	127.7(5)	C4-C8-C9	125.8(5)
O1-C9-O2	121.6(5)	O1-C9-C8	125.4(5)
O2-C9-C8	113.0(5)	C11-C10-H69A	109.5
C11-C10-H69B	109.5	H69A-C10-H69B	109.5
C11-C10-H69C	109.5	H69A-C10-H69C	109.5
H69B-C10-H69C	109.5	O2-C11-C10	108.6(5)
O2-C11-H68A	110.0	C10-C11-H68A	110.0
O2-C11-H68B	110.0	C10-C11-H68B	110.0
H68A-C11-H68B	108.3	N1-C12-C13	123.6(5)
N1-C12-C8	125.3(5)	C13-C12-C8	111.1(5)
C12-C13-C5	106.9(4)	C12-C13-C14	122.8(5)
C5-C13-C14	130.3(6)	O3-C14-O4	120.1(8)
O3-C14-C13	125.2(6)	O4-C14-C13	114.1(9)
O3-C14-O4A	124.7(9)	C13-C14-O4A	109.4(8)
N1-C17-Cr1	175.3(5)	O8-C18-Cr1	177.2(5)
O7-C19-Cr1	177.2(5)	O6-C20-Cr1	178.1(6)
O9-C21-Cr1	178.8(6)	O5-C22-Cr1	179.8(6)
C24-C23-C28	119.2(5)	C24-C23-P1	122.4(4)
C28-C23-P1	118.4(4)	C25-C24-C23	120.6(5)
C25-C24-H15	119.7	C23-C24-H15	119.7
C26-C25-C24	120.1(5)	C26-C25-H14	119.9
C24-C25-H14	119.9	C25-C26-C27	120.2(5)
C25-C26-H13	119.9	C27-C26-H13	119.9

C28-C27-C26	119.6(5)	C28-C27-H27	120.2
C26-C27-H27	120.2	C27-C28-C23	120.2(5)
C27-C28-H28	119.9	C23-C28-H28	119.9
C30-C29-C34	118.9(4)	C30-C29-P1	121.8(4)
C34-C29-P1	119.0(4)	C31-C30-C29	120.4(5)
C31-C30-H30	119.8	C29-C30-H30	119.8
C32-C31-C30	120.1(5)	C32-C31-H8	119.9
C30-C31-H8	119.9	C31-C32-C33	120.0(5)
C31-C32-H9	120.0	C33-C32-H9	120.0
C34-C33-C32	120.2(5)	C34-C33-H10	119.9
C32-C33-H10	119.9	C33-C34-C29	120.3(5)
C33-C34-H11	119.9	C29-C34-H11	119.9
C36-C35-C40	119.2(5)	C36-C35-P1	122.1(4)
C40-C35-P1	118.7(4)	C35-C36-C37	120.7(5)
C35-C36-H6	119.6	C37-C36-H6	119.6
C38-C37-C36	119.7(6)	C38-C37-H5	120.2
C36-C37-H5	120.2	C37-C38-C39	120.5(6)
C37-C38-H4	119.7	C39-C38-H4	119.7
C40-C39-C38	119.5(6)	C40-C39-H3	120.3
C38-C39-H3	120.3	C39-C40-C35	120.4(5)
C39-C40-H2	119.8	C35-C40-H2	119.8
C47-C41-C42	124.8(4)	C47-C41-S2	120.2(4)
C42-C41-S2	115.0(4)	C43-C42-C41	130.9(5)
C43-C42-H16	114.6	C41-C42-H16	114.6
C42-C43-C44	130.2(5)	C42-C43-H17	114.9
C44-C43-H17	114.9	C43-C44-C48	127.3(4)
C43-C44-C45	125.6(4)	C48-C44-C45	107.0(4)
C46-C45-C53	124.5(4)	C46-C45-C44	127.2(4)
C53-C45-C44	108.2(4)	C47-C46-C45	130.4(5)
C47-C46-H35	114.8	C45-C46-H35	114.8
C46-C47-C41	130.1(5)	C46-C47-H36	114.9
C41-C47-H36	114.9	C52-C48-C44	106.9(4)
C52-C48-C49	122.0(4)	C44-C48-C49	131.1(5)
O10-C49-O11	121.9(4)	O10-C49-C48	123.8(5)
O11-C49-C48	114.3(4)	C51A-C50-O11	107.3(7)
O11-C50-C51	108.6(7)	O11-C50-H20A	110.0
C51-C50-H20A	110.0	O11-C50-H20B	110.0
C51-C50-H20B	110.0	H20A-C50-H20B	108.4
C50-C51-H51A	109.5	C50-C51-H51B	109.5
H51A-C51-H51B	109.5	C50-C51-H51C	109.5

H51A-C51-H51C	109.5	H51B-C51-H51C	109.5
C50-C51A-H51D	109.5	C50-C51A-H51E	109.5
H51D-C51A-H51E	109.5	C50-C51A-H51F	109.5
H51D-C51A-H51F	109.5	H51E-C51A-H51F	109.5
N2-C52-C48	124.1(4)	N2-C52-C53	124.3(4)
C48-C52-C53	111.6(4)	C52-C53-C45	106.3(4)
C52-C53-C54	127.9(4)	C45-C53-C54	125.9(4)
O12-C54-O13	122.5(5)	O12-C54-C53	125.5(5)
O13-C54-C53	111.9(4)	O13-C55-C56	110.6(6)
O13-C55-H32A	109.5	C56-C55-H32A	109.5
O13-C55-H32B	109.5	C56-C55-H32B	109.5
H32A-C55-H32B	108.1	C55-C56-H33A	109.5
C55-C56-H33B	109.5	H33A-C56-H33B	109.5
C55-C56-H33C	109.5	H33A-C56-H33C	109.5
H33B-C56-H33C	109.5	N2-C57-Cr2	177.7(5)
O18-C58-Cr2	178.6(5)	O17-C59-Cr2	174.5(5)
O16-C60-Cr2	177.8(5)	O15-C61-Cr2	177.3(5)
O14-C62-Cr2	178.8(6)	C64-C63-C68	118.7(5)
C64-C63-P2	123.1(4)	C68-C63-P2	118.2(4)
C65-C64-C63	121.2(6)	C65-C64-H42	119.4
C63-C64-H42	119.4	C66-C65-C64	119.8(6)
C66-C65-H41	120.1	C64-C65-H41	120.1
C65-C66-C67	120.4(6)	C65-C66-H40	119.8
C67-C66-H40	119.8	C68-C67-C66	119.8(5)
C68-C67-H39	120.1	C66-C67-H39	120.1
C67-C68-C63	120.0(6)	C67-C68-H38	120.0
C63-C68-H38	120.0	C74-C69-C70	119.2(4)
C74-C69-P2	119.3(4)	C70-C69-P2	121.4(4)
C71-C70-C69	119.8(5)	C71-C70-H44	120.1
C69-C70-H44	120.1	C72-C71-C70	120.2(5)
C72-C71-H45	119.9	C70-C71-H45	119.9
C71-C72-C73	120.2(5)	C71-C72-H46	119.9
C73-C72-H46	119.9	C74-C73-C72	120.0(5)
C74-C73-H47	120.0	C72-C73-H47	120.0
C73-C74-C69	120.6(5)	C73-C74-H48	119.7
C69-C74-H48	119.7	C80-C75-C76	119.5(6)
C80-C75-P2	118.9(4)	C76-C75-P2	121.3(4)
C77-C76-C75	120.0(6)	C77-C76-H54	120.0
C75-C76-H54	120.0	C78-C77-C76	120.0(6)
C78-C77-H53	120.0	C76-C77-H53	120.0

C77-C78-C79	120.1(6)	C77-C78-H52	120.0
C79-C78-H52	120.0	C78-C79-C80	120.6(7)
C78-C79-H51	119.7	C80-C79-H51	119.7
C79-C80-C75	119.6(6)	C79-C80-H50	120.2
C75-C80-H50	120.2	Cl3S-C2S-Cl3S	124.0(7)
Cl3S-C2S-H2S1	106.3	Cl3S-C2S-H2S1	106.3
Cl3S-C2S-H2S2	106.3	Cl3S-C2S-H2S2	106.3
H2S1-C2S-H2S2	106.4	Cl1S-C1S-Cl2S	110.5(4)
Cl1S-C1S-Cl2A	102.8(6)	C2S-Cl3S-C2S	56.0(7)
C20-Cr1-C22	90.7(3)	C20-Cr1-C19	91.3(2)
C22-Cr1-C19	89.5(3)	C20-Cr1-C21	88.5(3)
C22-Cr1-C21	90.0(3)	C19-Cr1-C21	179.5(3)
C20-Cr1-C18	94.5(3)	C22-Cr1-C18	174.7(3)
C19-Cr1-C18	89.4(3)	C21-Cr1-C18	91.1(3)
C20-Cr1-C17	176.4(2)	C22-Cr1-C17	87.8(2)
C19-Cr1-C17	85.4(2)	C21-Cr1-C17	94.8(3)
C18-Cr1-C17	86.9(2)	C60-Cr2-C61	88.9(2)
C60-Cr2-C62	90.1(2)	C61-Cr2-C62	88.8(3)
C60-Cr2-C59	95.2(2)	C61-Cr2-C59	87.4(2)
C62-Cr2-C59	173.5(2)	C60-Cr2-C58	88.8(2)
C61-Cr2-C58	177.7(2)	C62-Cr2-C58	91.0(2)
C59-Cr2-C58	93.0(2)	C60-Cr2-C57	178.6(2)
C61-Cr2-C57	92.5(2)	C62-Cr2-C57	89.8(2)
C59-Cr2-C57	85.1(2)	C58-Cr2-C57	89.8(2)
C17-N1-C12	172.6(6)	C57-N2-C52	173.3(5)
C9-O2-C11	115.6(4)	C49-O11-C50	114.9(4)
C54-O13-C55	116.4(4)	C29-P1-C23	103.7(2)
C29-P1-C35	105.1(2)	C23-P1-C35	106.0(2)
C29-P1-Au1	117.43(17)	C23-P1-Au1	107.67(16)
C35-P1-Au1	115.69(17)	C75-P2-C63	105.4(3)
C75-P2-C69	104.2(2)	C63-P2-C69	105.0(2)
C75-P2-Au2	115.61(18)	C63-P2-Au2	107.85(17)
C69-P2-Au2	117.75(17)	C1-S1-Au1	108.07(18)
C41-S2-Au2	107.45(17)	C14-O4-C15	118.5(13)
C16-C15-O4	104.6(11)	C16-C15-H15A	110.8
O4-C15-H15A	110.8	C16-C15-H15B	110.8
O4-C15-H15B	110.8	H15A-C15-H15B	108.9
C15-C16-H16A	109.5	C15-C16-H16B	109.5
H16A-C16-H16B	109.5	C15-C16-H16C	109.5
H16A-C16-H16C	109.5	H16B-C16-H16C	109.5

C15A-O4A-C14	115.0(14)	O4A-C15A-C16A	113.7(15)
O4A-C15A-H15C	108.8	C16A-C15A-H15C	108.8
O4A-C15A-H15D	108.8	C16A-C15A-H15D	108.8
H15C-C15A-H15D	107.7	C15A-C16A-H16D	109.5
C15A-C16A-H16E	109.5	H16D-C16A-H16E	109.5
C15A-C16A-H16F	109.5	H16D-C16A-H16F	109.5
H16E-C16A-H16F	109.5		

Table S5. Torsion angles [°] for **8**·$^{3}/_{4}CH_2Cl_2$.

C7-C1-C2-C3	-10.1(9)	S1-C1-C2-C3	169.4(4)
C1-C2-C3-C4	2.6(10)	C2-C3-C4-C8	-176.0(5)
C2-C3-C4-C5	7.3(9)	C3-C4-C5-C6	-5.5(8)
C8-C4-C5-C6	177.3(5)	C3-C4-C5-C13	176.0(5)
C8-C4-C5-C13	-1.3(6)	C13-C5-C6-C7	175.1(5)
C4-C5-C6-C7	-3.2(9)	C5-C6-C7-C1	4.1(10)
C2-C1-C7-C6	5.0(9)	S1-C1-C7-C6	-174.5(5)
C3-C4-C8-C12	-175.7(5)	C5-C4-C8-C12	1.6(6)
C3-C4-C8-C9	7.2(9)	C5-C4-C8-C9	-175.5(5)
C12-C8-C9-O1	-177.1(6)	C4-C8-C9-O1	-0.6(9)
C12-C8-C9-O2	0.2(8)	C4-C8-C9-O2	176.7(5)
C4-C8-C12-N1	177.7(5)	C9-C8-C12-N1	-5.3(9)
C4-C8-C12-C13	-1.4(6)	C9-C8-C12-C13	175.7(5)
N1-C12-C13-C5	-178.5(5)	C8-C12-C13-C5	0.6(6)
N1-C12-C13-C14	1.4(8)	C8-C12-C13-C14	-179.6(5)
C6-C5-C13-C12	-178.1(5)	C4-C5-C13-C12	0.4(6)
C6-C5-C13-C14	2.1(9)	C4-C5-C13-C14	-179.4(5)
C12-C13-C14-O3	5.1(9)	C5-C13-C14-O3	-175.2(6)
C12-C13-C14-O4	175.8(9)	C5-C13-C14-O4	-4.4(11)
C12-C13-C14-O4A	-165.2(10)	C5-C13-C14-O4A	14.6(11)
C28-C23-C24-C25	1.9(8)	P1-C23-C24-C25	-176.8(4)
C23-C24-C25-C26	-1.1(8)	C24-C25-C26-C27	-0.5(8)
C25-C26-C27-C28	1.3(8)	C26-C27-C28-C23	-0.5(8)
C24-C23-C28-C27	-1.1(8)	P1-C23-C28-C27	177.7(4)
C34-C29-C30-C31	-0.6(8)	P1-C29-C30-C31	174.3(4)
C29-C30-C31-C32	-0.4(8)	C30-C31-C32-C33	1.0(8)
C31-C32-C33-C34	-0.6(9)	C32-C33-C34-C29	-0.3(8)
C30-C29-C34-C33	0.9(8)	P1-C29-C34-C33	-174.1(4)
C40-C35-C36-C37	-1.3(7)	P1-C35-C36-C37	177.1(4)
C35-C36-C37-C38	-0.6(8)	C36-C37-C38-C39	0.9(8)
C37-C38-C39-C40	0.7(9)	C38-C39-C40-C35	-2.5(8)
C36-C35-C40-C39	2.8(8)	P1-C35-C40-C39	-175.6(4)
C47-C41-C42-C43	4.3(9)	S2-C41-C42-C43	-174.7(5)
C41-C42-C43-C44	3.5(10)	C42-C43-C44-C48	175.0(5)
C42-C43-C44-C45	-2.3(9)	C43-C44-C45-C46	-6.2(8)
C48-C44-C45-C46	176.0(5)	C43-C44-C45-C53	176.2(5)
C48-C44-C45-C53	-1.6(5)	C53-C45-C46-C47	-174.4(5)
C44-C45-C46-C47	8.4(9)	C45-C46-C47-C41	0.8(10)

C42-C41-C47-C46	-8.2(9)	S2-C41-C47-C46	170.6(5)
C43-C44-C48-C52	-176.2(5)	C45-C44-C48-C52	1.5(5)
C43-C44-C48-C49	4.0(9)	C45-C44-C48-C49	-178.3(5)
C52-C48-C49-O10	-0.7(8)	C44-C48-C49-O10	179.1(5)
C52-C48-C49-O11	-179.1(4)	C44-C48-C49-O11	0.7(8)
C44-C48-C52-N2	178.0(4)	C49-C48-C52-N2	-2.2(7)
C44-C48-C52-C53	-0.9(6)	C49-C48-C52-C53	178.9(4)
N2-C52-C53-C45	-179.0(4)	C48-C52-C53-C45	-0.1(6)
N2-C52-C53-C54	0.6(8)	C48-C52-C53-C54	179.5(5)
C46-C45-C53-C52	-176.7(5)	C44-C45-C53-C52	1.0(5)
C46-C45-C53-C54	3.7(8)	C44-C45-C53-C54	-178.6(5)
C52-C53-C54-O12	175.4(5)	C45-C53-C54-O12	-5.0(9)
C52-C53-C54-O13	-3.5(7)	C45-C53-C54-O13	176.1(5)
C68-C63-C64-C65	0.4(10)	P2-C63-C64-C65	-176.3(5)
C63-C64-C65-C66	0.3(11)	C64-C65-C66-C67	-2.2(11)
C65-C66-C67-C68	3.4(10)	C66-C67-C68-C63	-2.7(9)
C64-C63-C68-C67	0.8(9)	P2-C63-C68-C67	177.6(5)
C74-C69-C70-C71	-1.5(8)	P2-C69-C70-C71	174.8(4)
C69-C70-C71-C72	0.2(8)	C70-C71-C72-C73	0.8(8)
C71-C72-C73-C74	-0.5(9)	C72-C73-C74-C69	-0.8(9)
C70-C69-C74-C73	1.8(8)	P2-C69-C74-C73	-174.6(4)
C80-C75-C76-C77	-1.2(8)	P2-C75-C76-C77	172.2(4)
C75-C76-C77-C78	-1.4(8)	C76-C77-C78-C79	2.1(9)
C77-C78-C79-C80	-0.2(10)	C78-C79-C80-C75	-2.4(9)
C76-C75-C80-C79	3.0(8)	P2-C75-C80-C79	-170.4(4)
Cl3S-C2S-Cl3S-C2S	0.0010(10)	O1-C9-O2-C11	2.2(8)
C8-C9-O2-C11	-175.2(5)	C10-C11-O2-C9	176.5(5)
O10-C49-O11-C50	-1.0(7)	C48-C49-O11-C50	177.4(4)
C51A-C50-O11-C49	166.6(14)	C51-C50-O11-C49	-164.0(15)
O12-C54-O13-C55	3.0(8)	C53-C54-O13-C55	-178.0(5)
C56-C55-O13-C54	81.3(7)	C30-C29-P1-C23	-100.6(4)
C34-C29-P1-C23	74.2(4)	C30-C29-P1-C35	10.5(5)
C34-C29-P1-C35	-174.6(4)	C30-C29-P1-Au1	140.8(4)
C34-C29-P1-Au1	-44.4(5)	C24-C23-P1-C29	14.8(5)
C28-C23-P1-C29	-164.0(4)	C24-C23-P1-C35	-95.7(5)
C28-C23-P1-C35	85.6(4)	C24-C23-P1-Au1	139.9(4)
C28-C23-P1-Au1	-38.8(4)	C36-C35-P1-C29	-95.6(4)
C40-C35-P1-C29	82.8(4)	C36-C35-P1-C23	13.9(5)
C40-C35-P1-C23	-167.7(4)	C36-C35-P1-Au1	133.1(4)
C40-C35-P1-Au1	-48.5(4)	C80-C75-P2-C63	-160.4(4)

C76-C75-P2-C63	26.2(5)	C80-C75-P2-C69	89.3(4)
C76-C75-P2-C69	-84.0(5)	C80-C75-P2-Au2	-41.5(5)
C76-C75-P2-Au2	145.2(4)	C64-C63-P2-C75	-115.9(5)
C68-C63-P2-C75	67.4(5)	C64-C63-P2-C69	-6.3(6)
C68-C63-P2-C69	177.0(5)	C64-C63-P2-Au2	120.1(5)
C68-C63-P2-Au2	-56.6(5)	C74-C69-P2-C75	-171.5(4)
C70-C69-P2-C75	12.2(5)	C74-C69-P2-C63	78.0(5)
C70-C69-P2-C63	-98.3(5)	C74-C69-P2-Au2	-41.9(5)
C70-C69-P2-Au2	141.8(4)	C2-C1-S1-Au1	10.9(5)
C7-C1-S1-Au1	-169.5(3)	C47-C41-S2-Au2	11.3(5)
C42-C41-S2-Au2	-169.7(3)	O3-C14-O4-C15	-6.3(17)
C13-C14-O4-C15	-177.6(10)	C14-O4-C15-C16	-178.0(16)
O3-C14-O4A-C15A	12.(2)	C13-C14-O4A-C15A	-177.4(13)
C14-O4A-C15A-C16A	89.(3)		

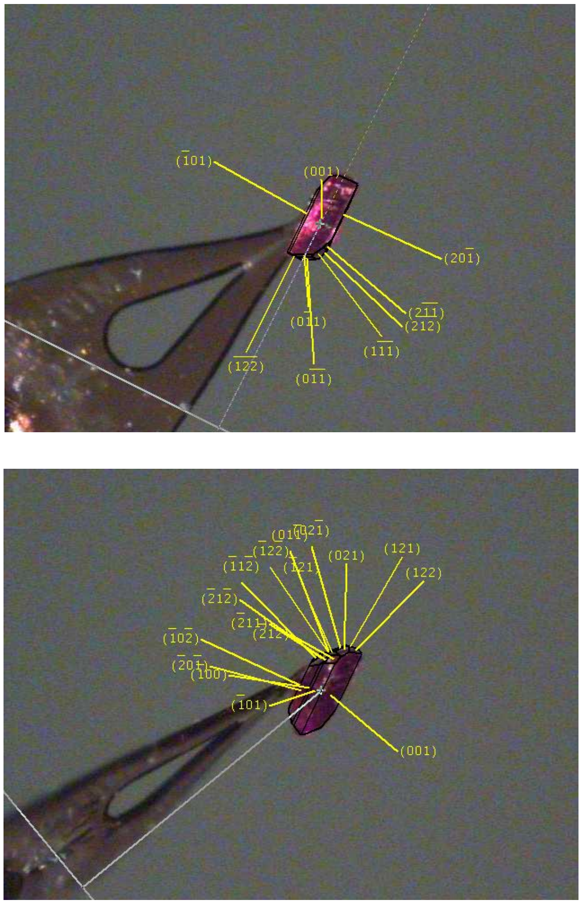

Figure S2. Photographs of the actual crystal of **8**·$^{3}/_{4}CH_2Cl_2$ used in the X-ray diffraction study that show crystal face indexing used for the absorption correction (SADABS).

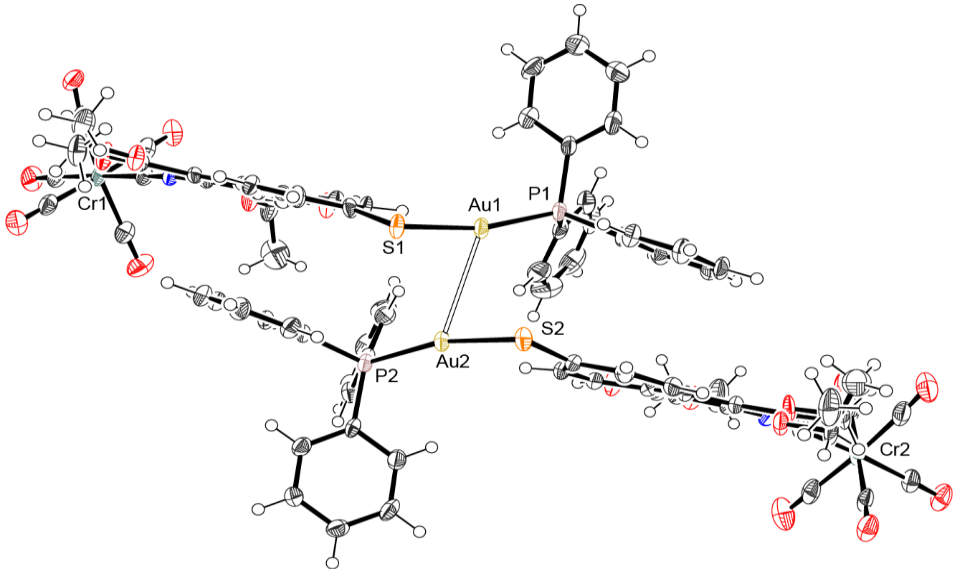

Figure S3. ORTEP diagram (50% thermal ellipsoids) of the asymmetric unit of **8**·$^{3}/_{4}CH_2Cl_2$ emphasizing aurophilic and face-centered Ph···azulenyl interactions between two crystallographically independent molecules of **8**. The disordered CH_2Cl_2 molecules or crystallization are omitted for clarity.

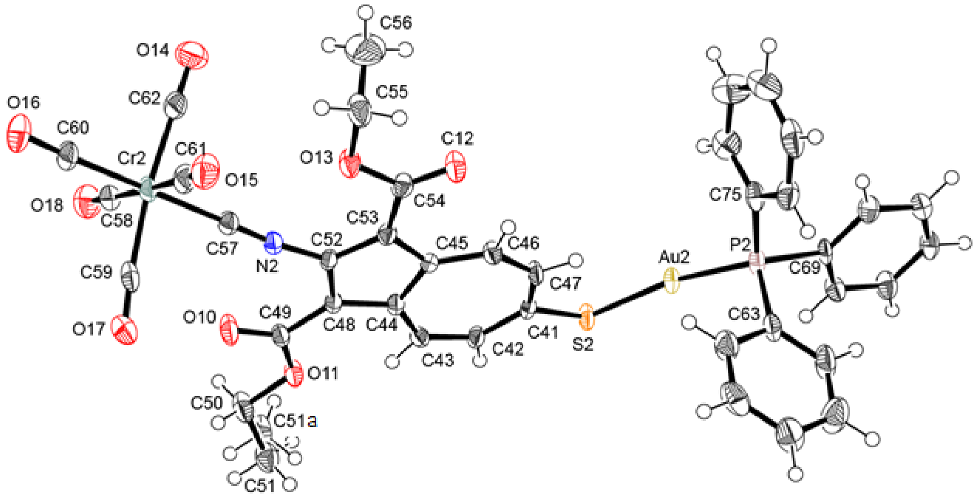

Figure S4. ORTEP diagram (50% thermal ellipsoids) of one of the two crystallographically independent molecules of **8** in the asymmetric unit. See Figure 3 of the main article for a thermal ellipsoid plot of the other molecule. Selected interatomic distances (Å) and angles (°): Au2-P2 2.274(1), Au2-S2 2.323(1), S2-C41 1.752(5), Cr2-C57 1.969(5), Cr2-C58 1.918(7), Cr2-C59 1.910(6), Cr2-C60 1.887(5), Cr2-C61 1.898(6), Cr2-C62 1.900(6), C57-N2 1.158(6), C58-O18 1.135(7), C59-O17 1.135(6), C60-O16 1.139(6), C61-O15 1.141(6), C62-O14 1.143(6), P2-Au2-S2 167.25(5), Au2-S2-C41 107.5(2), C52-N2-C57 173.3(5), Cr2-C57-N2 177.7(5).

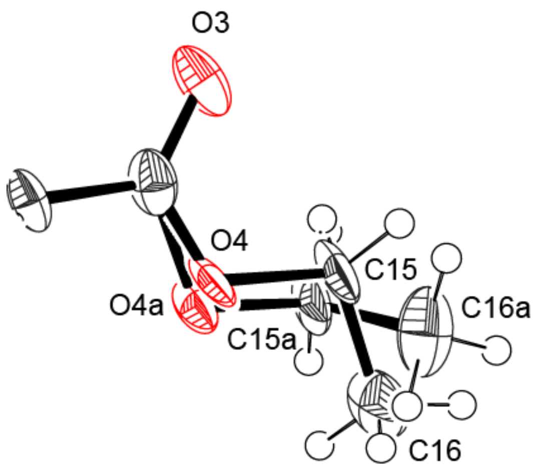

Figure S5. Detailed ORTEP drawings of the models used to resolve disordered ester groups in **8**·$^{3}/_{4}CH_2Cl_2$.

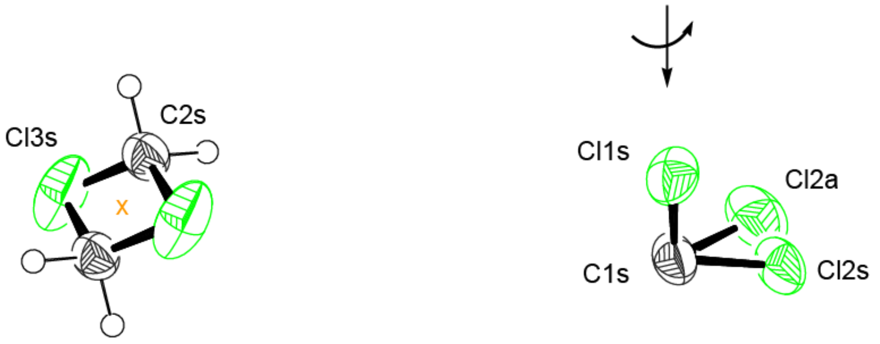

Figure S6. Left: ORTEP drawing of the disordered CH_2Cl_2 molecule of crystallization in **8**·$^{3}/_{4}CH_2Cl_2$ located at a special position (inversion center). Right: : ORTEP drawing of the CH_2Cl_2 molecule of crystallization in **8**·$^{3}/_{4}CH_2Cl_2$ disordered via rotation around the C1S-Cl1S bond; the H-atoms could not be attached to the carbon atom C1S in a meaningful way and were left out from the refinement.

C. SURFACE STUDIES

C1. Self-assembled monolayer films of 7, 1-3-diethoxycarbonyl-2-mercaptoazulene, and 1,3-diethoxycarbonyl-6-mercaptoazulene on the Au(111) surface. Commercial gold-coated silicon substrates (Platypus Technologies) featuring (111) preferred orientation normal to the substrate[7] were soaked sequentially in distilled chloroform, acetone, and 200-proof ethanol for two hours in each solvent. The bare gold substrates were thoroughly dried under a stream of N_2 gas and their ellipsometric physical constants *n* and *k* were measured. SAM films of these compounds were formed by placing a freshly cleaned ~1 × 1 cm^2 gold substrate into a 2 mM solution of either **7**, 6-mercapto-1,3-diethoxycarbonylazulene, or 6-mercapto-2-chloro-1,3-diethoxycarbonylazulene in $CHCl_3$ for *ca.* 24 hrs. Prior to their analysis, the SAM-coated substrates were rinsed thoroughly with $CHCl_3$ and dried in a flow of N_2 gas. No precautions to exclude air or ambient laboratory lighting were exercised during these SAM preparation experiments.

C2. Optical ellipsometry. The film thickness values were determined using an Auto EL III ellipsometer (Rudolph Research). All measurements were conducted with a HeNe laser at a wavelength of 632.8 nm and an incident angle of 70° to the surface normal. The optical constants *n* and *k* were obtained for each sample individually by measuring these parameters for the corresponding freshly cleaned bare gold substrates prior to the SAM formation. These optical constants were used as input in determining thicknesses of the adsorbed thin layers of **7**, 6-mercapto-1,3-diethoxycarbonylazulene, and 6-mercapto-2-chloro-1,3-diethoxycarbonylazulene. A refractive index of 1.45 was assumed[8] for all organic thin films described herein. For each SAM sample, the reported thickness value constitutes an average of those derived from ellipsometric measurements at five different spots of the SAM substrate.

C3. Surface IR measurements. The grazing incidence Reflection Absorption Fourier Transform Infrared Spectroscopy spectra for the SAMs of **7,** 1,3-diethoxycarbonyl-2-mercaptoazulene, and 1,3-diethoxycarbonyl-6-mercaptoazulene on gold were recorded using a Thermo Nicolet Nexus 670 FTIR spectrometer with a VeeMax grazing angle accessory set at an angle of 70°. A background spectrum was collected using a freshly cleaned bare gold substrate before acquiring the spectrum of each sample.One thousand scans from 600 to 4000 cm^{-1} at 4 cm^{-1} resolution were collected for each background/sample combination.

D. DFT AND TD-DFT CALCULATIONS

D1. Experimental

All Density Functional Theory (DFT) calculations were performed using the ORCA (v.2.9.1) program.[9] Geometric optimizations for **7** employed the BP86 functional[10] with a TZVP (Alrichs triple-ζ valence polarized)[11] basis set. The resolution of identity approximation (RI) was used along with the SV/J auxiliary basis set[12] and the Zero-Order Regular Approximation (ZORA).[13] Single point energy and time-dependent DFT (TD-DFT) calculations were subsequently performed employing the B3LYP functional,[14] a TZVP basis set, and a TZV/J auxiliary basis set.[12] Geometric optimizations, single point energy calculations, and TD-DFT calculations for the strictly organic molecules were performed using the B3LYP functional with a TZVP basis set, the RIJCOSX approximation, and a TZV/J auxiliary basis set.[12,14,15] The TD-DFT analysis of **7** involved calculating the first 100 lowest energy excited states.

For **7**, the solvation effects of dichloromethane (dielectric constant of 9.08 F/m) were modeled using the conductor-like screening model (COSMO), as implemented in ORCA.[16] Molecular and orbital images were produced with the aid of the gOpenMol (v.3.00) program with isodensity values set at ±0.03. The final Cartesian coordinates for all optimized structures considered herein are provided in Tables S7-S13. The UV-Vis absorption spectrum of **7** was simulated using an in-house developed program based on Eq. 1:

$$\varepsilon(\nu) = \frac{D_i\,\nu_i}{4*2.296\text{E}-39*\sqrt{\pi}*\sigma}\exp[-\left(\frac{\nu-\nu_i}{\sigma}\right)^2] \quad \textbf{(1)}$$

where ε is the molar absorptivity at ν - the energy in question; ν_i is the energy that each oscillator (f_i) contributes to ν; σ is the standard deviation of the Gaussian curve; D_i is defined by Eq. 2:

$$D_i = \frac{f_i}{4.7017556079\text{ E}29*\nu_i} \quad \textbf{(2)}$$

The geometry of complex (MeNC)Cr$(CO)_5$ in the gas phase was optimized using the BP86 functional[9] with a TZVP (Alrichs triple-ζ valence polarized) [10] basis set with the tight SCF convergence criteria. The energies and intensities of the ν_{CO} and ν_{NC} vibrations for this complex were calculated using the NumFreq option. IR spectra were generated using the orca_mapspc program provided by the ORCA software package.[8]

Table S6. Cartesian coordinates (Å) for the optimized structure of **7**.

Atom	x	y	z
C	0.197319	-0.290037	-0.055776
O	0.317606	1.137435	-0.357349
C	1.559606	1.673437	-0.205308
C	1.613129	3.103614	-0.558895
C	0.533567	4.006140	-0.807198
N	-0.782724	3.681615	-0.724847
C	-1.905421	3.314347	-0.638636
C	1.036657	5.292942	-1.163235
C	2.460080	5.203356	-1.146481
C	3.343481	6.256659	-1.440708
C	4.732983	6.285978	-1.442420
C	5.651785	5.259666	-1.141175
C	5.356028	3.936174	-0.761137
C	4.118291	3.323946	-0.593798
C	2.820751	3.839172	-0.751618
H	4.145903	2.278567	-0.279005
H	6.213890	3.287118	-0.566469
S	7.355837	5.776111	-1.277131
H	7.915171	4.590065	-0.915142
H	5.172158	7.249434	-1.716706
H	2.849904	7.188511	-1.724754
C	0.288412	6.520634	-1.492130
O	-1.016230	6.475269	-1.108466
C	-1.782836	7.686504	-1.408472
C	-3.144230	7.554611	-0.760295
H	-3.730881	8.462810	-0.964966
H	-3.699293	6.697114	-1.161298
H	-3.058088	7.441243	0.328698
H	-1.224921	8.552747	-1.025968
H	-1.850716	7.788621	-2.501564
O	0.780653	7.503275	-2.039342
O	2.525595	1.017992	0.175740
C	0.534981	-1.143864	-1.267167
H	0.355871	-2.203333	-1.027569
H	-0.094983	-0.877009	-2.126323
H	1.591185	-1.032555	-1.546056
H	0.850027	-0.523981	0.795380
H	-0.850552	-0.407769	0.243564
Cr	-3.748517	2.677270	-0.502608
C	-3.236330	1.563794	0.952079

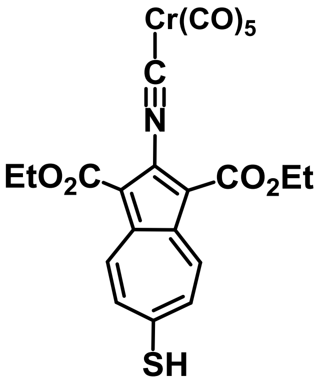

O	-2.932488	0.881698	1.836469
C	-3.330651	1.273171	-1.718243
O	-3.085835	0.416129	-2.456115
C	-5.530427	2.041941	-0.375754
O	-6.619008	1.653354	-0.298223
C	-4.240361	3.792179	-1.964835
O	-4.546004	4.460682	-2.858649
C	-4.155991	4.073391	0.728930
O	-4.414956	4.906791	1.488030

Table S7. Cartesian coordinates (Å) for the optimized structure of **7** with implicit CH_2Cl_2 solvent.

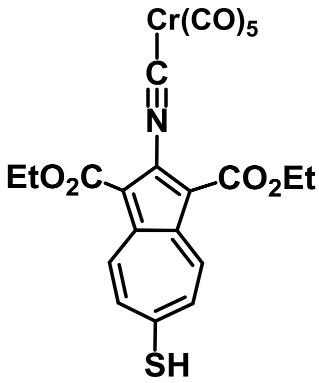

Atom	x	y	z
C	0.221142	-0.328695	-0.111367
O	0.360660	1.089172	-0.463113
C	1.525766	1.689514	-0.109606
C	1.586158	3.099560	-0.528511
C	0.504374	3.979066	-0.828970
N	-0.812110	3.647668	-0.748207
C	-1.940585	3.301729	-0.667455
C	1.002214	5.250432	-1.235580
C	2.426389	5.174285	-1.199191
C	3.304130	6.227751	-1.512269
C	4.692030	6.271043	-1.493539
C	5.614755	5.258272	-1.153314
C	5.327388	3.941823	-0.738427
C	4.092128	3.330342	-0.557091
C	2.792209	3.834641	-0.736646
H	4.133675	2.297215	-0.207992
H	6.188384	3.305025	-0.522676
S	7.313529	5.777814	-1.286410
H	7.883456	4.607914	-0.889536
H	5.126056	7.230663	-1.786899
H	2.815824	7.150567	-1.830622
C	0.238993	6.446310	-1.631405
O	-1.001140	6.489598	-1.084982
C	-1.808272	7.664728	-1.437544
C	-3.032935	7.672719	-0.548396
H	-3.651062	8.547307	-0.798936
H	-3.642875	6.771958	-0.696106
H	-2.753279	7.741783	0.511985
H	-1.192802	8.563041	-1.294036
H	-2.068595	7.592195	-2.503718
O	0.677746	7.333479	-2.363358
O	2.432451	1.103763	0.481672
C	0.817521	-1.227514	-1.181227
H	0.613612	-2.277734	-0.922445
H	0.368173	-1.025419	-2.163876
H	1.905741	-1.096623	-1.251847
H	0.689593	-0.496743	0.866897
H	-0.862009	-0.471267	-0.025102
Cr	-3.806119	2.706523	-0.545249

C	-3.316224	1.485938	0.826780
O	-3.030905	0.739734	1.665119
C	-3.451353	1.375449	-1.858211
O	-3.246857	0.562331	-2.656290
C	-5.600599	2.122495	-0.438277
O	-6.701996	1.763356	-0.372971
C	-4.279407	3.930800	-1.922096
O	-4.577116	4.665894	-2.765678
C	-4.156055	4.022521	0.785517
O	-4.382374	4.804539	1.608257

Table S8. Energies and the corresponding oscillator strengths of the first 100 transitions from the TD-DFT calculations on **7** in CH_2Cl_2 solution.

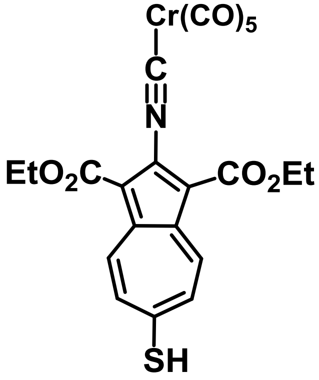

State	Energy (cm^{-1})	F oscillator
1	21050.6	0.008440933
2	19548.6	0.008302915
3	21566.9	0.440930158
4	20993	0.000142998
5	25529.2	0.031783777
6	29175.1	0.000523999
7	23145.8	8.74E-05
8	24658.5	0.00153919
9	31650.4	0.176097303
10	32233.8	0.027514476
11	32309.1	0.004273783
12	32584.2	0.024114703
13	31489.5	0.011731022
14	32961.2	0.004396496
15	33430.6	0.377010195
16	33856.2	0.200372532
17	34120.5	0.167133858
18	33703.7	0.000715809
19	33437.6	0.004133436
20	34520.8	0.639317963
21	34245.9	0.022829785
22	34497.4	0.003954268
23	35767.7	0.005262534
24	35749.6	0.184225884
25	36236.3	0.000253981
26	36266.8	2.73E-06
27	37235	5.99E-06
28	37385.4	0.000139643
29	37451.5	0.002606689
30	37744.6	6.92E-06
31	37249.6	0.004730236
32	37829.9	0.01455088
33	32003.1	0.000230613
34	38145	0.023432543
35	37575.9	0.043338614
36	32991.4	2.68E-05
37	38612.1	0.005880548
38	38899.4	0.003155376
39	39268.3	0.04478096

40	36209.6	0.014066849
41	37318.5	0.033356435
42	37520.2	0.053271894
43	40004.2	0.007654526
44	40428.4	0.270487457
45	40190.4	0.165763047
46	40725.1	0.016156875
47	40875	0.072881788
48	41071.6	0.087421929
49	41057.3	0.114699011
50	37345.2	0.001050751
51	41431.9	0.001260359
52	42134.2	0.000505756
53	40410.6	6.87E-05
54	35984.9	3.22E-05
55	40751.4	0.015278054
56	42359.7	0.070006684
57	38394.4	0.000299784
58	43422.6	0.001980381
59	43138.4	0.000662857
60	43964.4	0.010503904
61	40034.6	0.020223888
62	38904.5	7.34E-05
63	41613.3	0.026564704
64	43390.8	0.017351242
65	42179.1	0.067419722
66	42922.5	0.001304344
67	45918.1	0.00018831
68	46118.8	0.002075025
69	45105.5	0.035488195
70	46096.2	0.155933638
71	45636.9	0.093714283
72	46918.9	0.000279326
73	47398.6	0.009065992
74	41364	3.76E-05
75	47603.6	0.000665109
76	47602.7	0.001841715
77	47066.5	0.07956117
78	39316.9	0.005179794
79	42580.7	0.004257019
80	47808.6	0.016334654
81	42124.3	7.03E-05
82	48325.1	0.000598739
83	48614.3	0.090759572
84	44974	0.025208489

85	48998.1	0.001313656
86	48590.7	0.001283855
87	48868.2	0.012498906
88	49264.2	0.066067226
89	49604.2	0.00117
90	45038.6	0.016333071
91	39323.9	0.000361772
92	45211.5	0.009660361
93	49683.4	0.163278528
94	47599.1	0.184006995
95	47034.1	0.060441672
96	46635.2	0.004456557
97	50732	0.016578008
98	47942	0.054456003
99	48422.5	0.021668848
100	50193.3	0.083783143

Table S9. TD-DFT-calculated optical transitions for the lower energy portion of the electronic excitation spectrum of **7**.

Calc. Transition (nm)	Calc. Transition w/ 35nm Offset (nm)	Oscillator Strength	Composition (%)	Experimental Transition (nm)
415	452	0.9201	84.92 % HOMO→LUMO	452
			5.74 % HOMO-1→LUMO+1	
			4.80 % HOMO-3→LUMO	
318	355	0.1003	82.5 % HOMO-3→LUMO+1	360
			9.83 % HOMO-1→LUMO+1	
			1.25 % HOMO→LUMO+1	
		0.3397	56.42 % HOMO→LUMO+2	
			17.39 % HOMO-1→LUMO+1	
			7.03 % HOMO-3→LUMO+1	
			3.87 % HOMO-6→LUMO	
			3.36 % HOMO-3→LUMO+2	
			2.53 % HOMO-2→LUMO+6	
			1.65 % HOMO→LUMO+7	
			1.64 % HOMO-5→LUMO	
			1.57 % HOMO-3→LUMO+7	
		0.665	41.77 % HOMO-1→LUMO+1	
			27.75 % HOMO→LUMO+2	
			5.23 % HOMO-3→LUMO+1	
			4.13 % HOMO-6→LUMO	
			3.21 % HOMO-5→LUMO	
			3.04 % HOMO→LUMO+5	
			2.93 % HOMO→LUMO	
			1.90 % HOMO-2→LUMO+6	
			1.08 % HOMO→LUMO+7	
			1.08% HOMO-3→LUMO+2	
			1.02 % HOMO-3→LUMO+5	
			1.01 % HOMO-12→LUMO+1	

Table S10. Cartesian coordinates (Å) for the optimized structure of **8a**.

Atom	x	y	z
Cr	0.157725	-0.581924	-0.199254
C	0.827670	0.899149	-1.287015
N	1.232838	1.793775	-1.944608
C	1.701710	2.825046	-2.695118
C	3.070243	3.019198	-3.049287
C	4.228176	2.176576	-2.717139
O	3.873885	1.003256	-2.114349
C	4.985089	0.144395	-1.763396
H	5.576563	-0.105515	-2.653915
H	5.634927	0.637012	-1.027569
H	4.529994	-0.754455	-1.337765
O	5.399437	2.459805	-2.950136
C	3.132659	4.217568	-3.825726
C	1.780448	4.746048	-3.946115
C	0.898595	3.874665	-3.232928
C	-0.552418	4.067573	-3.113254
O	-1.144660	3.145773	-2.297640
C	-2.577198	3.290761	-2.159225
H	-3.073605	3.174240	-3.131720
H	-2.828428	4.276254	-1.744700
H	-2.881062	2.495034	-1.472359
O	-1.199446	4.952281	-3.668454
C	1.392230	5.914102	-4.630475
C	2.146364	6.828289	-5.350883
C	3.536852	6.859404	-5.612392
C	4.482950	5.910972	-5.147991
C	4.305334	4.772252	-4.377132
H	5.212566	4.208073	-4.151524
H	5.515258	6.106862	-5.449405
S	4.262639	8.156422	-6.581634
Au	2.572842	9.581477	-7.330770
P	1.020542	11.068906	-8.147230
C	-0.310422	10.304935	-9.177183
H	0.142821	9.787559	-10.032058
H	-0.857559	9.566028	-8.577722
H	-1.009072	11.072221	-9.539399
C	0.098497	12.012764	-6.853031
H	0.810147	12.577960	-6.237386
H	-0.619696	12.706061	-7.314158
H	-0.438940	11.311029	-6.201987

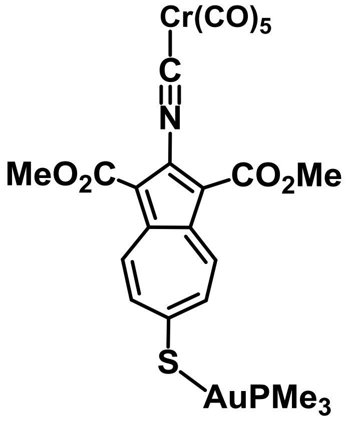

C	1.737369	12.379244	-9.235961
H	2.485343	12.951710	-8.672665
H	2.235355	11.909258	-10.093329
H	0.950666	13.057576	-9.595328
H	1.579802	7.655544	-5.787768
H	0.319905	6.118770	-4.583759
C	-0.488446	-2.012227	0.850719
O	-0.886334	-2.890182	1.495503
C	-1.255795	-0.830039	-1.442730
O	-2.120967	-0.984155	-2.196206
C	1.245455	-1.783945	-1.187029
O	1.906781	-2.527210	-1.778956
C	1.584241	-0.305669	1.024584
O	2.451685	-0.142575	1.773120
C	-0.921144	0.637933	0.775641
O	-1.584220	1.376951	1.371137

Table S11. Energies and the corresponding oscillator strengths of the first 100 transitions from the TD-DFT calculations on **8a**.

State	Energy (cm^{-1})	F oscillator
1	22078.9	0.009393924
2	24028	0.920071787
3	24471.3	0.0009623
4	26653.1	0.000200122
5	27359.8	0.029340936
6	28213.8	0.10580217
7	30046	0.000018273
8	30363.5	0.000211018
9	30571.3	0.100353703
10	31119.8	0.339768443
11	31776.4	0.665028378
12	32051.2	0.000022633
13	32076.3	0.000111182
14	32378.4	0.004712191
15	32911.7	0.000572353
16	33010	0.011018298
17	33131.2	0.009961105
18	33141.8	0.002239243
19	33997.9	0.001571266
20	34013.1	0.000300542
21	34264.4	0.017162359
22	34710.2	0.000070992
23	34797	0.000355311
24	34859.8	0.000575744
25	35519.3	0.004063054
26	35646.4	0.107827905
27	35807.7	0.009646971
28	35901.9	0.025487274
29	36383.8	0.082141059
30	36763.4	0.00054062
31	36821.1	0.000015305
32	36955.3	0.000135661
33	37055.6	0.00010389
34	37074.1	0.001792136
35	37322	0.000004073
36	37533.2	0.000553645
37	37605.4	0.000089527
38	37948.8	0.000006251

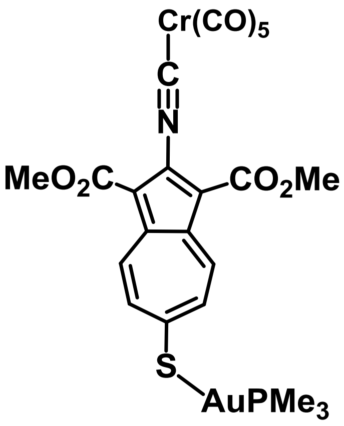

39	38005.4	0.002188903
40	38026.2	0.000824465
41	38050.6	0.002097961
42	38091.1	0.018890906
43	38117.4	0.001941848
44	38306.4	0.000256588
45	38539.1	0.261688496
46	38604.9	0.000094711
47	38910.8	0.000025234
48	38954.7	0.000021126
49	39174.8	0.045108853
50	39408.9	0.006141457
51	39585	0.004179797
52	39611.4	0.494978754
53	39915.4	0.161624594
54	39957.2	0.01509874
55	40193.7	0.128950322
56	40386	0.007918139
57	40665.9	0.024779098
58	40778.1	0.100872743
59	40848.7	0.000258864
60	40980.4	0.000425405
61	41115.4	0.006398719
62	41148.2	0.001658406
63	41253.2	0.029377315
64	41290	0.047108782
65	41704.1	0.000353646
66	41958.9	0.00678232
67	42016.4	0.000229202
68	42195.7	0.00013244
69	42206.1	0.002655544
70	42459.4	0.003419835
71	42501	0.000069598
72	42696.7	0.000004462
73	42738.1	0.020233518
74	42979.2	0.000108571
75	43388.3	0.000037254
76	43715.4	0.000082619
77	44072.6	0.000012799
78	44108.7	0.000020406
79	44329	0.000536452
80	44510.1	0.014139102
81	44590.3	0.0537072
82	44841.8	0.003006592
83	44862.6	0.000400914

84	45120.1	0.008975067
85	45356.6	0.074080203
86	45512.2	0.088101517
87	45698.7	0.000282297
88	45807.4	0.086152013
89	45841	0.000945418
90	45902.2	0.00007152
91	46026.5	0.091526773
92	46145.2	0.000932258
93	46231.5	0.004590593
94	46298.7	0.072063391
95	46349.8	0.041378363
96	46474.7	0.00504141
97	46575.4	0.002212128
98	46745	0.020858921
99	46842.3	0.011174769
100	46874.7	0.000017203

Table S12. TD-DFT-calculated optical transitions for the lower energy portion of the calculated electronic excitation spectrum of **8a**.

Calc. Transition (nm)	Oscillator Strength	Composition (%)	Experimental Transition for 8 in CH_2Cl_2 (nm)
464	0.4409	84.44 % HOMO→LUMO	469
		6.27 % HOMO-1→LUMO+1	
		4.09 % HOMO-4→LUMO	
		2.74 % HOMO-2→LUMO	
	0.0083	93.52 % HOMO-2→LUMO	
		2.76 % HOMO-2→LUMO+2	
		2.58 % HOMO→LUMO	
292	0.377	30.50 % HOMO-3→LUMO+3	333
		18.10 % HOMO→LUMO+5	
		14.44 % HOMO-2→LUMO+4	
		7.76 % HOMO-1→LUMO+1	
		6.01 % HOMO-2→LUMO+2	
		5.40 % HOMO→LUMO+2	
		2.94 % HOMO→LUMO+4	
		2.92 % HOMO-4→LUMO	
		2.32 % HOMO-4→LUMO+5	
		1.97 % HOMO-7→LUMO	
		1.84 % HOMO-5→LUMO	
		1.65 % HOMO→LUMO	
	0.2004	58.96 % HOMO-3→LUMO+3	
		17.79 % HOMO→LUMO+5	
		4.60 % HOMO-1→LUMO+1	
		3.93 % HOMO-2→LUMO+4	
		3.57 % HOMO-5→LUMO	
		2.59 % HOMO-4→LUMO	
		2.30 % HOMO-4→LUMO+5	
		1.37 % HOMO-7→LUMO	
	0.1671	36.11 % HOMO→LUMO+5	
		22.53 % HOMO-2→LUMO+4	
		16.47 % HOMO-5→LUMO	
		6.99 % HOMO→LUMO+2	
		5.41 % HOMO-1→LUMO+1	
		3.52 % HOMO-4→LUMO+5	
		1.38 % HOMO-4→LUMO	
		1.11 % HOMO-6→LUMO+1	
		1.09 % HOMO→LUMO	

	1.09 % HOMO→LUMO+8
0.0007	51.90 % HOMO-5→LUMO
	32.25 % HOMO-2→LUMO+4
	2.99 % HOMO→LUMO+2
	2.84% HOMO-2→LUMO+7
	2.08 % HOMO-3→LUMO+3
	1.49 % HOMO→LUMO+5
	1.07 % HOMO-6→LUMO+1
0.0041	52.84 % HOMO-2→LUMO+5
	35.70 % HOMO-2→LUMO+2
	4.30 % HOMO-2→LUMO+4
	2.11 % HOMO-2→LUMO+12
	1.36 % HOMO→LUMO+4
0.6393	21.94 % HOMO-5→LUMO
	21.14 % HOMO-1→LUMO+1
	11.93 % HOMO-4→LUMO
	10.56 % HOMO→LUMO+2
	7.21 % HOMO-2→LUMO+4
	6.82 % HOMO-7→LUMO
	3.81 % HOMO→LUMO
	2.99 % HOMO-2→LUMO+7
	2.52 % HOMO-6→LUMO
	2.13 % HOMO-10→LUMO+1
	1.27 % HOMO-2→LUMO+2
	1.05 % HOMO-2→LUMO+5
0.0228	54.12 % HOMO-6→LUMO
	18.76 % HOMO-1→LUMO+2
	16.23 % HOMO-7→LUMO
	5.07 % HOMO-5→LUMO+1
0.004	59.8 % HOMO-3→LUMO+2
	19.37 % HOMO-2→LUMO+6
	4.53 % HOMO→LUMO+3
	4.16 % HOMO→LUMO+6
	3.37 % HOMO-3→LUMO+4
	2.72 % HOMO-3→LUMO+5
	1.50 % HOMO-3→LUMO

Table S13. Cartesian coordinates (Å) for the optimized structure of azulene.

Atom	x	y	z
C	-0.005342	-0.006744	0.004805
H	0.912824	-0.575501	-0.018636
C	-1.304721	-0.531462	0.010988
C	-2.240137	0.510560	0.042209
C	-1.548645	1.731847	0.057129
C	-0.088454	1.393413	0.032518
C	0.981896	2.277209	0.035749
C	0.967989	3.670837	0.062107
C	-0.129649	4.529782	0.092498
C	-1.492866	4.240194	0.104630
C	-2.120772	2.995516	0.088980
H	-3.208031	3.013174	0.103978
H	-2.153818	5.099938	0.130718
H	0.116062	5.587527	0.109802
H	1.939692	4.153262	0.058887
H	1.965417	1.814211	0.013808
H	-3.315254	0.401706	0.053640
H	-1.552031	-1.584480	-0.006665

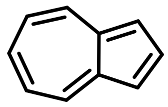

Table S14. Cartesian coordinates (Å) for the optimized structure of 1,3-diethoxycarbonyl-azulene.

Atom	x	y	z
C	0.079291	0.079207	-0.105621
O	-1.095790	0.922265	-0.078045
C	-2.291433	0.281259	-0.001764
C	-3.449701	1.186868	0.047084
C	-4.750250	0.673882	0.070960
C	-5.689591	1.709441	0.092362
C	-4.984242	2.945589	0.097633
C	-3.536010	2.608871	0.072325
C	-2.470874	3.506375	0.080843
C	-2.482391	4.898595	0.113656
C	-3.568290	5.767092	0.143660
C	-4.925601	5.463193	0.151385
C	-5.548610	4.218438	0.132349
H	-6.630036	4.233662	0.143592
H	-5.595773	6.315960	0.178163
H	-3.323227	6.824523	0.165600
H	-1.505471	5.370713	0.116431
H	-1.492357	3.047102	0.058375
C	-7.130369	1.410153	0.097016
O	-7.914465	2.509075	-0.057302
C	-9.344600	2.295776	-0.111457
C	-9.988668	3.652310	-0.314217
H	-9.615477	4.125625	-1.224538
H	-11.071548	3.542472	-0.405336
H	-9.781787	4.314541	0.528670
H	-9.567276	1.611257	-0.932111
H	-9.670403	1.820084	0.815449
O	-7.588188	0.294249	0.208353
H	-4.996235	-0.377108	0.064983
O	-2.375891	-0.927836	0.017922
C	1.294008	0.980797	-0.125081
H	2.199352	0.370361	-0.154867
H	1.291060	1.629661	-1.003332
H	1.334668	1.607890	0.768101
H	0.067913	-0.569962	0.771883
H	0.031670	-0.559795	-0.989610

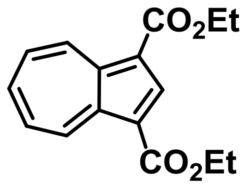

Table S15. Cartesian coordinates (Å) for the optimized structure of 2-isocyano-1,3-diethoxycarbonylazulene.

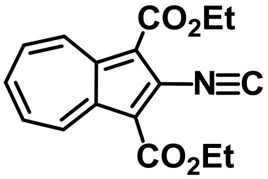

Atom	x	y	z
C	0.047815	0.081996	0.124313
O	-1.159256	0.882016	0.117535
C	-2.328652	0.189166	0.063931
C	-3.524429	1.053524	0.061525
C	-4.839563	0.533275	0.040239
C	-5.796855	1.574038	0.059961
C	-5.079785	2.801085	0.081293
C	-3.636550	2.470986	0.087178
C	-2.575541	3.378379	0.111675
C	-2.587750	4.769241	0.130373
C	-3.672596	5.638443	0.131293
C	-5.026915	5.325089	0.117090
C	-5.643182	4.078548	0.097463
H	-6.722962	4.094534	0.086841
H	-5.703747	6.172913	0.121994
H	-3.431202	6.696624	0.146697
H	-1.610156	5.239593	0.146492
H	-1.595974	2.924459	0.115854
C	-7.250997	1.317897	0.095930
O	-7.992285	2.447477	-0.058364
C	-9.431120	2.305590	0.020921
C	-10.013117	3.703745	0.062380
H	-9.756767	4.264339	-0.839255
H	-11.101602	3.652182	0.135329
H	-9.640078	4.253671	0.928670
H	-9.776745	1.742671	-0.848663
H	-9.682184	1.729473	0.912587
O	-7.754427	0.231040	0.252609
N	-5.145784	-0.791695	-0.012408
C	-5.406953	-1.934066	-0.070169
O	-2.355514	-1.018595	0.028502
C	1.227819	1.026857	0.171203
H	2.154256	0.448189	0.175432
H	1.241975	1.686244	-0.699198
H	1.206784	1.642923	1.072661
H	0.022098	-0.583068	0.989442
H	0.060745	-0.541262	-0.771399

Table S16. Cartesian coordinates (Å) for the optimized structure of 2-isocyano-6-mercapto-1,3-diethoxycarboylazulene (**3a**).

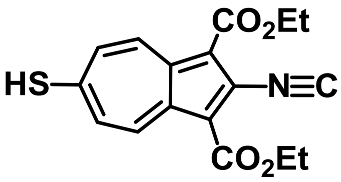

Atom	x	y	z
C	0.039290	0.069211	0.144582
O	-1.175240	0.857072	0.125400
C	-2.338876	0.152997	0.062131
C	-3.540528	1.008248	0.048625
C	-4.853281	0.482737	0.020557
C	-5.811630	1.524104	0.033442
C	-5.094265	2.749464	0.058296
C	-3.660801	2.425208	0.072983
C	-2.607289	3.342796	0.103928
C	-2.609067	4.728939	0.125839
C	-3.692060	5.612820	0.123502
C	-5.050947	5.277562	0.097184
C	-5.652681	4.032169	0.069257
H	-6.732179	4.051315	0.049886
H	-5.740491	6.115564	0.099122
S	-3.398092	7.373383	0.152368
H	-2.050191	7.326486	0.210877
H	-1.622930	5.179295	0.146223
H	-1.624598	2.894958	0.112364
C	-7.266662	1.274034	0.068968
O	-7.999014	2.410401	-0.082009
C	-9.437949	2.291053	0.020069
C	-9.991843	3.698302	0.118435
H	-9.748765	4.281155	-0.772731
H	-11.078736	3.666284	0.220319
H	-9.583802	4.213869	0.990027
H	-9.809616	1.763920	-0.861137
H	-9.682901	1.690482	0.896969
O	-7.776939	0.190688	0.225774
N	-5.156822	-0.842954	-0.030524
C	-5.414756	-1.986059	-0.088251
O	-2.354683	-1.054707	0.027233
C	1.209365	1.026128	0.199996
H	2.141630	0.457059	0.214973
H	1.225394	1.683218	-0.672191
H	1.173549	1.644352	1.099571
H	0.012812	-0.594154	1.010984
H	0.067169	-0.555596	-0.749670

Table S17. DFT-calculated (gas phase) and experimentally determined (IR, in *n*-hexane) ν_{NC} and ν_{CO} vibrational profile for C_{4v}-symmetric (MeNC)Cr(CO)$_5$.

	**This work (BP86/TZVP)**	**Ref. 17 (BP86/DZP)**	**Ref. 18 (experimental IR in *n*-hexane)**
$\nu_{NC}(A_1)$, cm^{-1}	2184	2177	2180
$\nu_{CO}(A_1^{(1)})$, cm^{-1}	2058	2045	2071
$\nu_{CO}(B_1)$, cm^{-1}	1992	1981	not IR-active
$\nu_{CO}(A_1^{(2)})$, cm^{-1}	1975	1966	1964
$\nu_{CO}(E)$, cm^{-1}	1969	1959	1964
$\nu_{CO}(E)$, cm^{-1}	1969	1959	1964

E. REFERENCES

1. T. C. Holovics, R. E. Robinson, E. C. Weintrob, M. Toriyama, G. H. Lushington and M. V. Barybin, *J. Am. Chem. Soc.*, 2006, **128**, 2300-2309.
2. D. L. DuBose, R. E. Robinson, T. C. Holovics, D. R. Moody, E. C. Weintrob, C. L. Berrie and M. V. Barybin, *Langmuir*, 2006, **22**, 4599-4606.
3. K. J. Scheetz, A. D. Spaeth, A. S. Vorushilov, D. R. Powell, V. W. Day and Mikhail V. Barybin, *Chem. Sci.*, 2013, **4**, 4267.
4. Software Package for Crystal Structure Solution, APEX 2. Bruker AXS, Madison, WI, 2010.
5. (a) R. H. Blessing, *Acta Cryst.*, 1995, **A51**, 33-38; (b) G. M. Sheldrick, SADABS Area-detector Absorption Correction, 2.03; University of Göttingen, Göttingen, Germany, 1999.
6. (a) L. J. Farrugia, *J. Appl. Cryst.*, 1997, **30**, 565; (b) M. N. Burnett; C. K. Johnson, ORTEP III: Report ORNL-6895; Oak Ridge National Laboratory: Oak Ridge, TN, 1996.
7. M. Aguilar, E. Anguiano, J. A. Aznárez, and J. L. Sacedón, *Surface Sci.*, 2001, **482-485**, 935-939.
8. (a) S. C. Clear and P. F. Nealey, *Langmuir*, 2001, **17**, 720-732; (b) J. D. Le Grange, J. L. Markham and C. R. Kurkjian, *Langmuir*, 1993, **9**, 1749-1753; (c) S. R. Wasserman, G. M. Whitesides, I. M. Tidswell, B. M. Ocko, P. S. Pershan and J. D. Axe, *J. Am. Chem. Soc.*, 1989, **111**, 5852-5861.
9. F. Neese, ORCA – an ab initio, Density Functional and Semiempirical Program Package, Version 2.9, University of Bonn, 2012.
10. (a) A. D. Becke, *J. Chem. Phys.*, 1986, **84**, 4524-4529. (b) J. P. Perdew, *Physical Review B*, 1986, **33**, 8822-8824.
11. (a) A. Schäfer, H. Horn and R. Ahlrichs, *J. Chem. Phys.*, 1992, **97**, 2571-2577. (b) A. Schäfer, C. Huber and R. Ahlrichs, *J. Chem. Phys.*, 1994, **100**, 5829-5835.
12. F. Neese, *J. Comput. Chem.*, 2003, **24**, 1740-1747.
13. D. A. Pantazis, X. Y. Chen, C. R. Landis and F. Neese, *J. Chem. Theory Comput.*, 2008, **4**, 908-919.
14. (a) A. D. Becke, *J. Chem. Phys*., 1993, **98**, 5648-5652; (b) A. D. Becke, *J. Chem. Phys*., 1993, **98**, 1372-1377; (c) C. Lee, W. Yang and R. G. Parr, *Phys. Rev. B*, 1988, **37**, 785-789.
15. R. Izsak and F. Neese, J. Chem. Phys., 2011, **135**, 144105.
16. S. Sinnecker, A. Rajendran, A. Klamt, M. Diedenhofen, and F. Neese, *J. Phys. Chem. A*, 2006, **110**, 2235–2245.
17. J. Wang, G. Li, Q. Li, Y. Xie and R. B. King, *Polyhedron*, 2012, **47**, 165.
18. J. A. Connor, E. M. Jones, G. K. McEwen, M. K. Lloyd and J. A. McCleverty, *J. Chem. Soc., Dalton Trans.*, **1972**, 1247.
